# Supplementary material for: ’[We] learned how to speak with love’: a qualitative exploration of accredited social health activist (ASHA) community health worker experiences of the Mobile Academy refresher training in Rajasthan, India
Source: BMJ Open. 2022 Jun 14;12(6):e050363. doi: 10.1136/bmjopen-2021-050363 (PMC9198783; doi:10.1136/bmjopen-2021-050363)
Supplement: Author's manuscript [file bmjopen-2021-050363.draft_revisions.pdf]

BMJ Open is committed to open peer review. As part of this commitment we make the peer review history of every article we publish publicly available.

When an article is published we post the peer reviewers' comments and the authors' responses online. We also post the versions of the paper that were used during peer review. These are the versions that the peer review comments apply to.

The versions of the paper that follow are the versions that were submitted during the peer review process. They are not the versions of record or the final published versions. They should not be cited or distributed as the published version of this manuscript.

BMJ Open is an open access journal and the full, final, typeset and author-corrected version of record of the manuscript is available on our site with no access controls, subscription charges or pay-per-view fees (http://bmjopen.bmj.com).

If you have any questions on BMJ Open's open peer review process please email info.bmjopen@bmj.com

BMJ Open

"[We] learned how to speak with love": a qualitative exploration of ASHA community health worker experiences of the Mobile Academy refresher training in Rajasthan, India

Journal:	*BMJ Open*
Manuscript ID	bmjopen-2021-050363
Article Type:	Original research
Date Submitted by the Author:	18-Feb-2021
Complete List of Authors:	Scott, Kerry; Johns Hopkins University Bloomberg School of Public Health, International Health<br>Ummer, Osama; Oxford Policy Management; BBC Media Action<br>Chamberlain, Sara; BBC Media Action<br>Sharma, Manjula; Independent researcher<br>Gharai, Dipanwita; Oxford Policy Management<br>Mishra, Bibha; Oxford Policy Management<br>Choudhury, Namrata; Jawaharlal Nehru University<br>LeFevre, Amnesty; Johns Hopkins University Bloomberg School of Public Health; University of Cape Town
Keywords:	Health policy < HEALTH SERVICES ADMINISTRATION & MANAGEMENT, MEDICAL EDUCATION & TRAINING, Human resource management < HEALTH SERVICES ADMINISTRATION & MANAGEMENT, Community child health < PAEDIATRICS, International health services < HEALTH SERVICES ADMINISTRATION & MANAGEMENT

SCHOLARONE™
Manuscripts

I, the Submitting Author has the right to grant and does grant on behalf of all authors of the Work (as defined in the below author licence), an exclusive licence and/or a non-exclusive licence for contributions from authors who are: i) UK Crown employees; ii) where BMJ has agreed a CC-BY licence shall apply, and/or iii) in accordance with the terms applicable for US Federal Government officers or employees acting as part of their official duties; on a worldwide, perpetual, irrevocable, royalty-free basis to BMJ Publishing Group Ltd ("BMJ") its licensees and where the relevant Journal is co-owned by BMJ to the co-owners of the Journal, to publish the Work in this journal and any other BMJ products and to exploit all rights, as set out in our licence.

The Submitting Author accepts and understands that any supply made under these terms is made by BMJ to the Submitting Author unless you are acting as an employee on behalf of your employer or a postgraduate student of an affiliated institution which is paying any applicable article publishing charge ("APC") for Open Access articles. Where the Submitting Author wishes to make the Work available on an Open Access basis (and intends to pay the relevant APC), the terms of reuse of such Open Access shall be governed by a Creative Commons licence – details of these licences and which Creative Commons licence will apply to this Work are set out in our licence referred to above.

Other than as permitted in any relevant BMJ Author's Self Archiving Policies, I confirm this Work has not been accepted for publication elsewhere, is not being considered for publication elsewhere and does not duplicate material already published. I confirm all authors consent to publication of this Work and authorise the granting of this licence.

For peer review only

"[We] learned how to speak with love": a qualitative exploration of ASHA community health worker experiences of the Mobile Academy refresher training in Rajasthan, India

Authors: Kerry Scott[1], Osama Ummer[2], Sara Chamberlain[3], Manjula Sharma[4], Dipanwita Gharai[2], Bibha Mishra[2], Namrata Choudhury[5], and Amnesty LeFevre[1,6]

[1] Department of International Health, Johns Hopkins Bloomberg School of Public Health, 615 N. Wolfe St, Baltimore, Maryland, USA
[2] Oxford Policy Management-Delhi, 4/6 First Floor, Siri Fort Institutional Area, New Delhi, Delhi 110049, India
[3] BBC Media Action, India, Innov8 Old Fort Saket District Mall, Saket District Centre, Sector 6, Pushp Vihar, New Delhi, Delhi 110017, India
[4] Independent researcher, New Delhi, India
[5] Centre for the Study of Law and Governance, Jawaharlal Nehru University
[6] School of Public Health and Family Medicine, University of Cape Town, Cape Town, South Africa

Corresponding author: Kerry Scott, 615 N. Wolfe St, Baltimore, MD, 21205, kscott26@jhu.edu, 647-675-7956.

Words in main text: 5236

Abstract

Introduction
Mobile Academy is a mobile-based training course for India's accredited social health activist (ASHA) community health workers. The course, which ASHAs access by dialling a number from their phones, totals four hours of audio content. It consists of 11 chapters, each with their own quiz, and provides a cumulative pass or fail score at the end. This qualitative exploration of Mobile Academy contributes to global understandings of how mLearning programs are experienced by frontline health workers and the potential of future mLearning programs.

Methods
We conducted in-depth interviews (n=25) and focus group discussions (n=5) with ASHAs and other health system actors. Open ended questions explored ASHA perspectives on Mobile Academy, the course's perceived influence on ASHAs, and preferences for future training programs. After applying *a priori* codes to the transcripts, we identified emergent themes and grouped them according to our mLearning for frontline health workers framework.

For peer review only - http://bmjopen.bmj.com/site/about/guidelines.xhtml

Results

ASHAs reported enjoying Mobile Academy, specifically praising its friendly tone and the ability to repeat content. They, and higher level health systems actors, conceived it to primarily be a test not a training. ASHAs reported that they found the quizzes easy but generally did not consider the course overly simplistic. ASHAs considered Mobile Academy's content to be a useful knowledge refresher but said its primary benefit was in modelling a positive communications approach, which inspired them to adopt a kinder, more "loving" communication style when speaking to beneficiaries. ASHAs and health system actors wanted follow-on mLearning courses that would continue to compliment but not replace face-to-face training.

Conclusion

This mLearning program for frontline health workers in India was well received by ASHAs across a wide range of education levels and experience. Dial-in audio training has the potential to not only reinforce topical knowledge but also showcase positive ways to communicate.

Keywords

mobile phones; mLearning; training and supervision; community health workers; frontline health workers; ASHAs; mHealth; mLearning; digital literacy; India

Strengths and limitations of this study

- This study presents user perspectives on Mobile Academy, a dial-in mobile phone based training program for accredited social health activist (ASHA) frontline health workers (FLHWs) in India.
- These perspectives are vital to understanding the potential of an mHealth training strategy that requires minimal supervisor input and can be used by FLHWs with low or no literacy, limited digital skills, and no smartphone or Internet access – common realities for community health workers in low resource settings.
- Our purposive sampling strategy using backend Mobile Academy data enables us to present insight into a variety of user experiences, including perspectives from typical users and a sub-set of atypically highly educated and digitally savvy ASHAs.
- Our findings highlight that Mobile Academy was well-received by community health workers across a range of educational levels, years of experience, and digital capabilities.
- We are limited to presenting the experiences of ASHAs in the Indian state of Rajasthan; given that Mobile Academy has been scaled up across 13 states, additional localized research is required.

For peer review only

Introduction

Frontline health workers (FLHWs), also called community health workers, have been widely recognised as an integral part of primary health care teams, particularly in low and middle income countries (LMICs).[1,2] Globally, FLHWs perform a range of health system functions to improve the acceptability and reach of health services, including counselling and health education, encouraging care-seeking and treatment adherence, and improving the reach of health care to marginalized communities.[3] However, FLHW performance is often hindered by a range of program and health system limitations, including insufficient training and supervision.[3,4]

With the rapid rise in mobile phone access and network connectivity in LMICs, the use of mobile technology for health (mHealth) has flourished.[5] mHealth strategies have been identified as one mechanism to improve FLHW training and support, although evidence of effectiveness is mixed, partially because of the diversity in interventions.[6–8] WhatsApp groups bolstered supportive supervision for Kenyan FLHWs[9] and motivational SMS messages improved the documentation of pregnancies by FLHWs in Malawi.[10] However, a human resources management tool called MOTECH did not improve perceived supervision, self-reported motivation, work engagement and job satisfaction among Sierra Leonean FLHWs.[11] Research from Bihar, India found that Mobile Kunji, a job aid for accredited social health worker (ASHA) community health workers and *anganwadi* nutrition workers, increased time spent with beneficiaries and improved beneficiary perceptions of health worker trustworthiness.[12] Women whose frontline health workers used Mobile Kunji had higher odds of registering their pregnancies, consuming iron-folic acid tablets, preparing for birth (saving money, planning transportation), exclusive breastfeeding and appropriate complementary feeding compared to their unexposed peers; no impact was identified on contraception or immunisation practices.[12] Continuum of Care Services, a job aid for India's three FLHWs (ASHAs, *anganwadis*, and auxiliary nurse midwives), increased the frequency of FLHW-beneficiary interactions and of FLHW coordination of home visits. It also improved FLHW job confidence and increased beneficiary practice of skin-to-skin care, immediate breastfeeding and appropriate complementary feeding.[13] It did not improve FLHW supervision or other health-related behaviors among beneficiaries.

While mHealth tools show potential as supervisory supports and job aids, little is known about digital platforms for FLHW training. This paper explores user and health system actor perceptions of Mobile Academy, a mobile phone-based training program, which was developed for India's FLHWs, including ASHAs. ASHAs are female community health workers who facilitate antenatal care, institutional deliveries, and immunizations, provide home-based newborn care, encourage family planning and convene village health events.[14,15] Current recruitment standards (released in 2012) dictate that ASHAs are required to have completed 10 years of formal education; ASHAs recruited in the first eight years of the program (2005 – 2012) required only 8 years' education and educational requirements can be "relaxed" in marginalized regions.[16] While some ASHAs have attained education beyond high school, non-literate ASHAs are not uncommon in many states[17]; 17% of ASHAs surveyed in Madhya Pradesh were unable to read a full sentence [18], and 30% of ASHAs surveyed in Uttar Pradesh were low literate.[19] State governments aim to provide ASHAs

For peer review only

with 23 days initial training and 10 days supplementary training, as well as monthly supportive supervision through the government primary healthcare system.[20] While program has been credited with bolstering antenatal care, increasing institutional deliveries[21–24] and childhood immunization[25,26] and improving community case management of childhood illness,[27–29] ASHAs have limited opportunities to refresh and expand their knowledge and receive insufficient supportive supervision.[29–33] Studies have consistently identified gaps in ASHA knowledge including on childhood illness and dangers signs in pregnancy.[27,31,34–36]

Mobile Academy, developed by BBC Media Action in India, provides ASHAs with refresher training on reproductive, maternal, neonatal and child health and seeks to help them better communicate with community members. The program consists of 44 short (2.5 minute) pre-recorded audio lessons (for a total of 240 minutes). The ASHAs answer 4 multiple choice quiz questions at the end of each of the 11 chapters (for a total of 44 questions) to reinforce the messages and build their confidence, including on using phones for learning. ASHAs dial in to a toll-free number to access the course and can listen to as much content as they like before ending the call. Bookmarking technology allows them to return to the lesson where they left off the next-time they call in. At the end of the course, ASHAs who score 50% or higher receive an SMS notifying them of their completion with a unique number; they can show this number to the primary health centre administration to receive a completion certificate. In half of the 13 states where Mobile Academy has been launched, 50% to 75% of all ASHAs have successfully completed the course. But in others states, uptake is below 15%.

While the program has been scaled up by the Government of India and completed by over 180,000 ASHAs to date, knowledge about user perceptions of Mobile Academy, its influence on interpersonal communication, and ASHA reflections on future mLearning opportunities remains limited. This paper presents the results of a qualitative study exploring these issues in Rajasthan, a state in northern India.

Methods

Study setting

Rajasthan is a Hindi-speaking state in western India with a population of 78 million.[37] While 85% of men are literate, literacy among women is only 57%.[38] The maternal mortality is 199 per 100,000 births[39] and the rural under-five mortality is 54.4 per 1000 births.[38] Only 39% of women receive the recommended four antenatal care checkups, but 84% of deliveries take place in health facilities.[38] As of January 2018, there were approximately 44,900 ASHAs in the state's rural areas, which is 88% of the targeted recruitment and amounts to 1 ASHA per 1147 rural people.

For peer review only - http://bmjopen.bmj.com/site/about/guidelines.xhtml

Sampling

We received permission to conduct this qualitative study in three districts (Ajmer, Sikar and Pali) of Rajasthan, selected by government authorities. Data collection took place in October and November 2018. ASHAs were selected purposively, using data generated by Mobile Academy's technical system and guidance from block-level ASHA facilitators. We interviewed ASHAs who had completed Mobile Academy when it was officially launched (2016, early 2017), as well as ASHAs who completed Mobile Academy more recently (late 2017, 2018). This decision was made to explore possible differences between early and late completers of Mobile Academy. We also hypothesized that late completers would be more likely to recall specific details of the course, since it would be fresher in their minds. We sought ASHAs with varied Mobile Academy engagement profiles. We considered the number of minutes an ASHA was engaged in the course (while most ASHAs in Rajasthan completed the course in approximately 240 minutes, we sought 11 ASHAs who took 270 minutes or more), their scores (while most ASHAs in Rajasthan had a score in the 40s out of a maximum of 44 points, we sampled 15 ASHAs with scores less than 40/44), and ASHAs who had repeated the course (we sampled four ASHAs who went through the course twice). Our FGDs included a mix of ASHAs by Mobile Academy completion data, nature of Mobile Academy engagement (quiz scores etc.) and education level. We also interviewed district and state level actors (Table 1).

Table 1. Mobile Academy respondent sample

Respondent	Male	Female	Total
In-depth interviews			
• ASHA	0	25	25
• ANM	0	6	6
• ASHA supervisor	3	3	6
• Block ASHA coordinator	2	1	3
• District actor	3	0	3
• State actor	1	1	2
Total	9	36	45
Focus group discussions			
• ASHAs (5 to 6 participants)	0	5	5

Recruitment, data collection and ethics

This study was approved by the institutional ethics review boards at Sigma, Delhi, India (10041/IRB/D/17-18) and JHU, Baltimore, USA (00008360). Interviews and FGDs were conducted by four female qualitative researchers (authors MS, DG, BM and NC), supported by a male research manager (OU) and female research coordinator (KS). All researchers were trained over a one-week period, which included pilot testing the detailed ASHA interview guide, and had master's level social science education or higher. They approached the respondents first by phone to explain the study, identify themselves as working for a Delhi-based research company, explain that they had governmental approval to conduct this study, and ask if they could meet face to face to learn more and, if they agreed, to participate. The interviews took about an hour and were conducted in ASHA homes and in

For peer review only - http://bmjopen.bmj.com/site/about/guidelines.xhtml

other stakeholder's offices and health facilities; the focus group discussions took just over an hour and were conducted in empty school buildings, clinics and courtyards. ASHA family members were often around the home during the ASHA interviews but did not actively listen to or engage with the interviews. Only the researchers and participants were present during the other data collection. The study information and informed consent was read to each potential participant and then summarized in conversational language to ensure comprehension. All respondents provided informed oral consent. Two people who we approached for the study refused: one ASHA who had a family emergency and one state level actor citing lack of interest. All respondents who agreed to participate in the study also allowed audio recording. The research domains explored are presented in box 1.

Box 1. Research domains explored

In-depth interviews

- Relationship with mobile technology (respondents' access to and use of mobile phones);
- Perception of Mobile Academy (voice, pace, content, relevance);
- Mobile Academy uptake (what motivated ASHAs to complete the course, how did they hear about it, did they encounter technological or social barriers to accessing it);
- Mobile Academy quizzes (difficulty, how respondent felt about them, use of the phone keypad to answer questions);
- Mobile Academy completion certificate (process for receiving certificate, importance of certificate);
- Extent to which Mobile Academy is a refresher training versus providing new content;
- Mobile Academy's influence on ASHA-beneficiary interaction and relationships (listening to Mobile Academy with others, telling others about Mobile Academy, drawing from Mobile Academy to help in communication with beneficiaries; the influence of Mobile Academy on ASHAs' confidence, respect and motivation);
- ASHA face-to-face trainings (content, benefits, drawbacks, relationships among ASHAs and between ASHAs and trainers, link to Mobile Academy);
- Desire for further training, especially through mobile technology (content, modality, dose)

Focus group discussions

- General perspectives of Mobile Academy;
- Challenges that ASHAs navigate in trying to promote birth preparedness and family planning;
- Whether Mobile Academy had any influence on how ASHAs spoke to people about these issues;
- ASHA perspectives on future mLearning.

Analysis

Analysis began with daily debrief meetings, wherein the field team (MS, DG, BM, NC, OU and KS) drew from interview notes to discuss emerging themes and adjust elements of the

For peer review only - http://bmjopen.bmj.com/site/about/guidelines.xhtml

guide and sampling based on saturation and emergent sub-topics without the study domains. After data collection, all audio files were transcribed and translated into English and uploaded into Dedoose, a qualitative data management software. Guided by the principles of thematic analysis, KS and OU developed and applied a coding framework with nine code clusters which echoed the topics explored in the interviews and FGDs. The clusters consisted of between three and 11 codes which were primarily drawn from *a priori* areas of interest.

After coding, we generated code reports that enabled us to read all text tagged by the same code. While reviewing these reports we were mindful of the respondent profiles and ASHA-ANM-ASHA facilitator clusters to identify any patterns or trends that existed. We present our findings thematically, grouped under the six components of our framework for understanding CHW engagement with mobile learning courses (Fig 1).

[Figure 1. Framework for understanding CHW engagement with mobile learning courses]

Patient and Public Involvement

The research was shaped by ASHA and other health system actor priorities, experiences and preferences through iterative probing and flexibility within our research domains. Results were disseminated to Government of India stakeholders and actors involved in developing Mobile Academy.

Findings

1. CHW profile

Mobile Academy was considered relevant and appropriate by ASHAs across a wide range of demographic and engagement profiles

While the ASHAs in our five focus groups were selected based only on having completed Mobile Academy and being geographically close to one another, the 25 ASHAs selected for interviews respondent sample was non-typical in several ways (table 2). We sought the rare ASHAs who completed Mobile Academy late (2018 or late 2017), in a state where 93% of ASHAs completed Mobile Academy in 2016 and early 2017. Our late completers (n=12) turned out to be newly recruited ASHAs who were also more educated than early completers (n=13).[16] This selection skewed our respondent sample towards ASHAs with higher education, which is not representative of ASHAs in the state. Whereas only 2 (8%) of the ASHAs interviewed in our study had eight or fewer years of education, over 70% of Rajasthan's ASHAs did.[20] However, our five focus group discussions were composed primarily of "typical" ASHAs (recruited into the ASHA program in the mid-2000s, fewer than 10 years of education, completed Mobile Academy early, and achieved high scores), which enabled us to round out the range of perspectives captured in this research.

For peer review only - http://bmjopen.bmj.com/site/about/guidelines.xhtml

Table 2. ASHA respondents interviewed in this study compared to ASHAs overall in Rajasthan state

Parameter	Our IDI sample n (%) (n=25)	Rajasthan N (%) (n= see notes)
Education		
<=8	2 (8%)	72%*
9 – 10	7 (28%)	23%*
11 – 12	11 (44%)	3%*
Graduate	5 (20%)	1%*
Mobile Academy		
Early completion (2016, early 2017)	13 (52%)	41,164 (93%)**
Late completion (late 2017, 2018)	12 (48%)	1,440 (3%)**
Average quiz score (out of 44 points)	31.6	42.6**
Failed (<22/44)	6 (24%)	91 (0.2%)**
Average duration (minutes)***	291.2	255.0#
ASHAs who repeated the course	3 (12%)	533 (13%)#

*From *ASHA Which Way Forward* (2011) based on a sample of 200 ASHAs
** All ASHAs who completed Mobile Academy in Rajasthan (n=44,545)
*** Of first course. If an ASHA repeated the course, this value would approximately double, but we are not considering this second attempt here, since we report repeats in a separate row.
# Only ASHAs who completed Mobile Academy in Sikar, Pali and Ajmer Districts

We did not identify a qualitative difference between early and late completers in terms of their recall of content or their interpretation of Mobile Academy's appropriateness and ease. Both groups reported that the course was a knowledge assessment and a refresher training, which covered the same content as their core face-to-face trainings. They reported that the course was relevant and appropriate, and easy to understand. ASHAs across the spectrum of education described the quizzes as easy and enjoyable.

We also sought ASHAs for interviews who had lower scores and who went through Mobile Academy more than once. We included six ASHAs who failed (24% of our sample) when only 0.2% of ASHAs in Rajasthan failed Mobile Academy. Surprisingly, the six ASHAs who failed told us that they unaware that they had performed poorly – despite the fact that the course informs users when they have failed – and, like the ASHAs with high scores, described the course as "easy". All three ASHAs who repeated Mobile Academy (ASHA_11, ASHA_12 and ASHA_24) told us that they did so because their supervisors told them that their first attempt was not properly logged in the system, and that they had to complete the course again. This explanation is likely a miscommunication or misunderstanding because our records show that each attempt was logged.

ASHAs in our sample had universal mobile phone access and variable digital literacy

All the ASHAs in our sample reported having their own phones, which they carried with them when they went out and could use without asking permission. In many households, the ASHA's phone was also used by other family members. Almost half the ASHAs in our sample reported that they used a smart phone, which indicates a more digitally-enabled group of ASHAs compared to the norm; in Madhya Pradesh a recent survey found that only 11% of ASHAs had a smartphone.[40] Even among our savvy qualitative sample, internet access was variable since few ASHAs bought mobile internet data regularly. (Internet access

For peer review only - http://bmjopen.bmj.com/site/about/guidelines.xhtml

had no bearing on Mobile Academy access because Mobile Academy is a dial-in mobile phone service.) ASHAs explained that the government-issued SIM for ASHA-related activities allowed them to speak to other ASHAs and ASHA facilitators and make calls to beneficiaries; however in some areas this government SIM did not work because of network issues or late payment of monthly bills by the government. ASHAs kept a second, personal, SIM, which they sometimes used for ASHA work as well.

Some ASHAs reported receiving calls from government actors, including their ANM and ASHA facilitator, as well as health system actors in district headquarters, the state capital and Delhi. These calls were to inform them about trainings and meetings, ask ASHAs to report data such as the number of pregnancies, or, in one case, to ask the ASHA to follow up with specific high risk patients. Others had not received any ASHA-work related phone calls. Over half of the ASHAs in this qualitative sample were on WhatsApp, compared to 12% in a representative sample of ASHAs in Madhya Pradesh.[40] Our ASHAs reported using WhatsApp to share reports and information with and among ASHAs, however access to WhatsApp was irregular and many ASHAs explained that their husbands were actually in the ASHA WhatsApp groups but reported information from the WhatsApp groups back to them.

All ASHAs were confident that they could receive calls from beneficiaries about emergencies, such as women in labor, and could make outgoing calls to the ambulance and to families, often to notify them about vaccinations and health checkups. Beyond receiving and making calls, most ASHAs self reported that they could open and read SMS and WhatsApp messages but several disclosed that they got stuck sometimes doing these activities. Our research team did not test these capacities. Most said that they were not able to write and send messages by SMS or in WhatsApp, even among those who completed education beyond 12th standard: "Neither do I know how to send SMS nor do I send. It is not required." (ASHA_08, 44/44, graduate). Several ASHAs described needing help from family members to operate their phones, including saving contacts and opening and reading messages.

2. Program access and uptake

Mobile Academy was widely understood to be primarily an examination

ASHAs and higher-level health system stakeholders described Mobile Academy as an ASHA knowledge test or examination rather than a refresher course. Relatedly, several higher level stakeholders (DIST_01, BLCK_01, BLCK_02) considered Mobile Academy to be an assessment of the ASHAs and were concerned that Mobile Academy could be completed by other people or that ASHAs could cheat by sharing the answers to the quizzes.

ASHAs completed Mobile Academy because of pressure from above, but also expressed desire to gain knowledge

ASHAs described pressure to complete Mobile Academy "*aage se* [from above]." They repeatedly reported being told by their supervisors that completing the course was *zaroori* [necessary/essential] and were also aware that the ANM and ASHA supervisor were under pressure from district level authorities to have all their ASHAs finish Mobile Academy.

For peer review only - http://bmjopen.bmj.com/site/about/guidelines.xhtml

> There was pressure, it's important to do it… we were getting messages, phone calls, again and again, that this is important for you to do. (FGD_ASHA_03)

A few ASHAs described being worried that they would lose their positions if they did not complete Mobile Academy or if they did not pass. One ASHA also reported being told that ASHA pay would increase upon completing Mobile Academy, which was not the case (FGD_ASHA_03).

In addition to being required to take the course, ASHAs described being motivated to completed Mobile Academy by their desire to gain more knowledge, as well as general curiosity, an interest in receiving the certificate and a desire to show higher level health system actors that they are knowledgeable.

> We get to learn, that it the reason. There is a will to learn, as an ASHA - for ourselves and for the villagers, like our family, like our village (ASHA_15, 43/44, 12th)

Mobile Academy uptake among our sample did not follow planned dose and frequency

While ASHAs could call into Mobile Academy and proceed through the course whenever they had time over weeks or even months, the ASHAs we spoke to reported completing it rapidly in just one or two days. National level data on Mobile Academy engagement found that 36% of ASHAs completed the course within three days, and 12% within the first 24 hours.[41] Our data can offer insight into what might have driven this sizable minority of ASHAs to proceed so rapidly. Some of the ASHAs in our sample reported not knowing that the course could be completed in parts and some were unsure how much time could elapse between starting and completing the course. Others worried about not being able to get through to the Mobile Academy line if they called back later. Many also emphasised that they were under pressure to complete the course quickly.

3. Experience of Mobile Academy

Initial fear replaced by happiness; ASHAs praised the pace and tone, valued repetition

When ASHAs first heard that they were to complete a training by mobile phone, they described feeling nervous. They did not know what it would entail or were concerned about having to provide answers to quiz questions. However, they quickly relaxed and enjoyed the course because of the warm and friendly tone, option to repeat content, and the fact that the quiz questions covered familiar topics and were easy.

> At first, I was very scared that I would give the wrong answer. Then when I finished it, I felt very good. (FGD_ASHA_05)

For peer review only

> I thought I do not know what she will ask and what she will not ask. This was the fear. But after listening I was not afraid. (ASHA_23, 38/44, 10th)

ASHAs expressed very positive overall opinions of the course. The character voices were frequently described as loving, warm and calm: "Sweetness was there in the voice" (ASHA_24, 44/44, 12th, early adopter). The Hindi language speech was easy to understand for the ASHAs, although they generally spoke Marwadi, a Rajasthani dialect close to Hindi.

> Her voice was good and sweet. […] She spoke exactly spoke like us. (ASHA_29, 35/44, 12th)

> We could trust her well. She was talking in such a way that it was nobody else but someone of her own, apna hi hai [like she knows us]. (ASHA_15, 43/44. 12th)

> They explained with a lot of love, very slowly. And asked [questions] calmly. (FGD_ASHA_03)

ASHAs felt the pace was appropriate: "neither it was fast nor slow; everything was explained in a proper manner" (ASHA_29) and greatly valued being able to repeat content. ASHAs emphasized that the repetitive content and ability to re-listen to lessons was a major strength of the program: "they were repeating some of the topics two to three times and then we were able to remember that nicely" (ASHA_31, 21/44, 8th).

Quizzes increased ASHA confidence

As designed, the ASHAs experienced quizzes to be easy but enjoyable. Although described as "easy" by all, ASHAs still said they had to pay attention properly in order to complete them.

> ASHA1: [I] got 100 out of 100. I felt like, if more like these are happening, we will do it.
> ASHA2: We had a lot of fun, lot of fun
> ASHA3: That we cleared the paper [passed the test]! If we get the next paper [test], we will clear that too.
> ASHA2: We passed it easily. We didn't have to listen to anyone scolding us.
> [all laugh]
> ASHA4: I felt like I passed a competition
> ASHA3: I was so happy to get good marks (FGD_ASHA_03)

> Absolutely we have to keep our mind on one point. Whatever they tell we have to find out the answer from that immediately. We must not delay. (ASHA_24, 44/44, 12th)

As mentioned above, those with low quiz scores were unaware that there was any issue with their score and described the quizzes as "easy". None reported receiving feedback that they had failed, despite the fact that the course reports back to users whenever they select an incorrect quiz answer and if they have failed at the end, suggesting that ASHAs forgot, did not realize, or did not want to admit to our research team that they had struggled with the quiz.

For peer review only - http://bmjopen.bmj.com/site/about/guidelines.xhtml

Mobile Academy is often a social activity

Despite the individual dial-in design, many ASHAs reported involving other people. Some (ASHA_31, FGD_ASHA_05) reported proceeding through Mobile Academy with a group of other ASHAs during group training sessions. Some played the content for colleagues, such as anganwadi workers and ASHA supervisors, to show them what the course was about or to seek assistance. Others played Mobile Academy on speakerphone for their children or in-laws to explain why they were on the phone for a long duration and to share the content.

Mobile Academy a complement to face-to-face training

ASHAs greatly valued that mobile phone-based training allowed them to complete the course from home, when they had spare time. This home-based model avoided the difficulties of making childcare arrangements and leaving behind domestic responsibilities inherent to face-to-face training or meetings.

> Otherwise [for face-to-face training] we have to leave the family and children and go. We have tension in that case. But here we can sit back at home and do the training. We can do it whenever we get time. (ASHA_03, 44/44, 10th)

Furthermore, ASHAs in remote areas valued that mLearning did not require the time and discomfort of travelling long distances to reach training centres. Two ASHAs (ASHA_11, ASHA_18) also noted that they could focus better alone at home compared to group settings where multiple people were speaking at once.

> We can listen to it peacefully. There, [face-to-face training] ten people are sitting. Who is saying what? Here we can listen comfortably. (ASHA_11, 42/44, 8th)

Several ASHAs contrasted the kind and encouraging approach of Mobile Academy with the punitive and demoralizing approach to training and supervision that they experienced face-to-face. ASHAs reported being frightened of supervisors and trainers, explaining that no ASHAs speak because “we feel a bit scared”, “if we are wrong he will scold us” (FGD_ASHA_03); “they scold us badly” (FGD_ASHA_05). However, other ASHAs found face-to-face trainers to be supportive and noted that in-person training offered the opportunity to ask questions whenever they did not understand; to learn techniques and practices by seeing them demonstrated; to connect with other ASHAs (“all the ASHAs get together, they talk and gossip” (FGD_ASHA_05)), trainers and facilitators; and to get out of the house.

> ASHA1: Sister, it’s like, in training face to face we will get to learn more. […] In Mobile Academy, we can only listen. In the other one, we will get to learn –
> ASHA2: The way/technique
> ASHA1: Practical
> ASHA3: They show us
> ASHA2: In face to face training, madam, if we ask questions we also get answers. We can ask also, what, why is this. In that [Mobile Academy], we can't even ask. We can just say yes or no. (FGD_ASHA_04)

Most ASHAs explained that both modalities of training had benefits and drawbacks, and thus hoped to receive further face-to-face training, supplemented with mLearning.

For peer review only - http://bmjopen.bmj.com/site/about/guidelines.xhtml

4. Mobile Academy's influence on ASHAs

Mobile Academy showcased a positive communication style that ASHAs can apply when speaking to beneficiaries

When asked about Mobile Academy's influence, ASHA most commonly explained that the course changed how they communicated with beneficiaries. They explained that they modelled the tone used by the voice actors in Mobile Academy to speak to beneficiaries, introducing a more loving and calm approach to their communication with women:

> [We] learned how to speak with *prem* [love] (laughs) […] After doing Mobile Academy, we've made some changes in the way we talk. […] This only: explain once. If they don't understand then explain again, but explain with love. Don't get angry, that won't work there. (FGD_ASHA_03)

They spoke of internalizing the importance of taking time to talk to beneficiaries, not rushing, and repeating content in a caring manner.

Mobile Academy increased ASHA confidence and reinforced knowledge

ASHAs reported feeling more confident and knowledgeable after completing Mobile Academy. As noted above, Mobile Academy was designed as a refresher training that covered the same topics as face-to-face training; ASHAs reported experiencing it in this way and said that the refresher content enabled them to retain some specific knowledge.

> If they tell us again, it makes a difference. […] We are able to understand [now] that the training happened for Mobile Academy. The course refreshes things. [Face-to-face] trainings happened here 8 to 10 years ago." (FGD_ASHA_03)

While ASHAs reported that they did not tell beneficiaries that they completed Mobile Academy and did not listen to Mobile Academy with beneficiaries, they drew from their training, including Mobile Academy, to answer beneficiary questions.

> Earlier we did not have courage to talk in front of others. Now we have courage. If we did not know anything, what would we tell others? If we have a little bit of knowledge only then we can tell others. If anybody asks anything, we can answer them as well. (ASHA_15, 43/44, 12th)

While ASHAs were to receive Mobile Academy completion certificates, many did not. ASHAs who had not received a certificate did not articulate personal disappointment or concern about community perception. However, some worried that they lacked proof to show health system stakeholders that they completed the course.

5. Barriers and facilitators facing ASHAs in their outreach and counselling activities

We explored barriers and facilitators for ASHAs when putting Mobile Academy's lessons into practice. By nature of Mobile Academy being a refresher mLearning program, its content

For peer review only - http://bmjopen.bmj.com/site/about/guidelines.xhtml

conveys the same information as is covered in face-to-face trainings. Thus, our findings present general challenges and opportunities that ASHAs experience while undertaking their counselling and outreach work, with a focus on birth preparedness and family planning.

ASHAs linked positive outcomes to increasing education levels among beneficiaries, multiple counselling visits that strengthened ASHA-beneficiary relationships, engaging mothers'-in-law, and improvements to services provided by the government. However ASHAs also spoke at length about the challenges they faced. Some ASHAs described having little shared identity with and low influence among the populations they serve. Some noted tension with beneficiaries and described the families that did not adhere to their guidance as stubborn, lazy and careless. Other ASHAs were sympathetic towards families that did not abide by their recommendations, particularly on birth preparedness, explaining that people were too poor to save money, too overworked to prepare, that husbands controlled the money, mothers-in-law resisted institutional birth, or families feared costs associated with accessing a hospital. ASHAs reported that beneficiaries avoid government facilities for childbirth because of poor quality care; being tasked with promoting government services in the face of quality constraints eroded beneficiary trust. In terms of encouraging the use of modern contraceptives to avoid or delay pregnancy, ASHAs faced pushback from community members who worried that contraception could harm them. ASHAs were also unable to counter persistent son preference: "No matter how many children they have, they will keep going until it's a boy" (FGD_ASHA_05). Enduring shame and secrecy around reproduction hindered frank discussion about contraception and careseeking among pregnant women.

6. Future training

ASHAs and higher-level health system actors were very positive about integrating future mLearning alongside face-to-face training to reinforce, refresh, or deepen understanding. ASHAs wanted future Mobile Academy content to cover newborn care, family planning, nutrition, non-communicable disease (diabetes, hypertension and cancer), adolescent health, village health committees, tuberculosis, malaria, leprosy, and cataracts. They said that future mobile training could consist of up to six hours of content, spread in half hour increments over several weeks or months.

Discussion

This qualitative exploration of Mobile Academy found strong positive perceptions of the course among ASHAs and other health system actors. ASHAs found Mobile Academy material relevant and appropriate, were driven to take the course because of a desire to learn and pressure from supervisors. They appreciated its warm and friendly tone and their ability to repeat content. ASHAs valued the course for refreshing and deepening their knowledge as well as showcasing a positive communication style that they could apply to interactions with beneficiaries. Respondents were enthusiastic about future mLearning, although they noted that face-to-face training could not be replaced with mobile phone-based programs.

For peer review only - http://bmjopen.bmj.com/site/about/guidelines.xhtml

In addition to these outcomes, several surprising aspects of ASHA engagement with the course emerged. First, although Mobile Academy was designed for ASHAs with eight years of education, ASHAs with 12 or more years of education also said that they valued the course. As ASHA educational requirements and mobile phone savvy increase, research examining whether some ASHAs can handle more complex material, alternative teaching styles, and the more sophisticated digital engagement used elsewhere will be valuable.[42–44] Nonetheless, given the persistence of low literacy and limited digital skills among women in India[45] including ASHAs[18,19,46], ongoing investment in audio based trainings that can be accessed from any phone, rather than textual or smartphone based programming, remains critical.

Second, although Mobile Academy was designed to be a training program with simple quiz questions that served only to motivate and engage ASHAs, the quiz component became the focus. Further research is needed to understand the range of effective ways to engage interactive feedback as motivators, teaching tools, and systems for assessment and accreditation. Third, the ASHAs in our sample tended to complete the course rapidly, in one or two long sessions, rather than spreading the content over many weeks. Nationally, a smaller portion (36%) took a rapid approach.[41] The ASHAs in our qualitative study explained that they would have liked to proceed through the course more slowly, but took the rapid approach because of pressure from supervisors and concern that they might be unable to access the course again later. This finding, as well as the outsized focus on the quizzes, suggests that supervisors and FLHWs require steady communication about the intent and engagement options for mLearning programs. Fourth, ASHAs and health system actors understood Mobile Academy as an internal accreditation rather than an achievement that could bolster community trust. Strengthening ASHA-beneficiary relationships will require ongoing ASHA capacity building, of which mLearning can play a role, community engagement, and quality improvements in the government health services themselves.[47]

Conclusion

While mLearning programs have been found capable of improving aspects of FLHW knowledge, patient engagement, and supervision, they tend to require smartphone technology and user digital skills that remain out of reach to many of India's rural FLHWs.[38,39] Many also rely on trainer inputs such as remote moderation and feedback[9], that are challenging add-ons in overstretched systems such as India's where vacancies throughout the supervisory hierarchy remain an issue.[48] Mobile Academy required only a basic phone and the ability to use a keypad to access its pre-recorded content and interactive quiz questions. It was well received by the ASHA FLHWs in our sample, which included a wide range of education levels and experience, including many ASHAs with more education and better digital access than the norm. Mobile Academy showcased the potential to reinforce knowledge and encourage positive beneficiary-FLHW communication style. These findings position the Mobile Academy model as a viable mLearning option for many LMIC contexts to reinforce ongoing face-to-face training.

For peer review only - http://bmjopen.bmj.com/site/about/guidelines.xhtml

Acknowledgements

The authors are grateful to the ASHAs and other health system actors who generously provided their time and insights to make this research possible. This work was made possible by the Bill and Melinda Gates Foundation. We thank Diva Dhar, Suhel Bidani, Rahul Mullick, Dr. Suneeta Krishnan, Dr. Neeta Goel and Dr. Priya Nanda for believing in us and giving us this opportunity. We additionally thank Vinit Pattnaik at OPM and Erica Crawford at Johns Hopkins for their support to the financial management of our work.

Data sharing

Data for this study consist of qualitative interview and focus group discussion transcripts. Uploading all transcripts for open availability would compromise our ability to fully mask participant details. However, we are happy to share anonymized portions of these transcripts upon reasonable request.

Competing interests

All authors have completed the Unified Competing Interest form (available on request from the corresponding author) and declare that the research reported was funded by the Bill and Melinda Gates Foundation. SC is employed by BBC Media Action, one of the entities supporting program implementation for Mobile Academy. The authors do not have other relationships and are not engaged in activities that could appear to have influenced the submitted work.

Contributor statement

KS, SC , OU and AEL conceptualized and designed the study. MS, DG, BM and NC conducted the data collection and preliminary data analysis through daily analytic debriefs. KS and OU led and managed the data collection and analysis, including the coding and thematic synthesis. KS drafted the manuscript and revised it based on critical and substantive input from SC, OU, AEL, MS, BM and NC. All authors agree to be accountable for all aspects of the work related to accuracy and integrity.

Funding statement

This work was supported by the Bill and Melinda Gates Foundation grant number OPP1179252.

Works Cited

1. WHO. The World Health Report 2008. Primary Health Care - Now more than ever [Internet]. Vol. 26, The World Health Report. Geneva, Switzerland; 2008. Available from: http://www.who.int/whr/2008/whr08_en.pdf
2. WHO. Global Strategy on Human Resources for Health: Workforce 2030 [Internet]. Synthesis Paper of the Thematic Working Groups. Geneva; 2016. Available from:

For peer review only

http://www.who.int/hrh/resources/pub_globstrathrh-2030/en/

3. Scott K, Beckham S, Gross M, Pariyo G, Rao K, Cometto G, et al. What do we know about community-based health programs? A systematic review of existing reviews on community health workers and their integration with health systems. Hum Resour Health. 2018;1–17.
4. Cometto G, Ford N, Pfaffman-Zambruni J, Akl EA, Lehmann U, McPake B, et al. Health policy and system support to optimise community health worker programmes: an abridged WHO guideline. Lancet Glob Heal [Internet]. 2018;6(12):e1397–404. Available from: http://dx.doi.org/10.1016/S2214-109X(18)30482-0
5. Marcolino MS, Oliveira JAQ, D'Agostino M, Ribeiro AL, Alkmim MBM, Novillo-Ortiz D. The Impact of mHealth Interventions: Systematic Review of Systematic Reviews. JMIR mHealth uHealth [Internet]. 2018 Jan 17;6(1):e23–e23. Available from: https://www.ncbi.nlm.nih.gov/pubmed/29343463
6. Agarwal S, Perry HB, Long LA, Labrique AB. Evidence on feasibility and effective use of mHealth strategies by frontline health workers in developing countries: Systematic review. Trop Med Int Heal. 2015;20(8):1003–14.
7. O'Donovan J, Bersin A, O'Donovan C. The effectiveness of mobile health (mHealth) technologies to train healthcare professionals in developing countries: a review of the literature. BMJ Innov [Internet]. 2015 Jan;1(1):33–6. Available from: http://innovations.bmj.com/lookup/doi/10.1136/bmjinnov-2014-000013
8. O'Donovan J, O'Donovan C, Kuhn I, Sachs SE, Winters N. Ongoing training of community health workers in low-income and middle-income countries: a systematic scoping review of the literature. BMJ Open [Internet]. 2018 Apr 1;8(4):e021467. Available from: http://bmjopen.bmj.com/content/8/4/e021467.abstract
9. Henry JV, Winters N, Lakati A, Oliver M, Geniets A, Mbae SM, et al. Enhancing the Supervision of Community Health Workers With WhatsApp Mobile Messaging: Qualitative Findings From 2 Low-Resource Settings in Kenya. Glob Heal Sci Pract [Internet]. 2016 Jun 20;4(2):311–25. Available from: http://www.ghspjournal.org/lookup/doi/10.9745/GHSP-D-15-00386
10. Joos O, Silva R, Amouzou A, Moulton LH, Perin J, Bryce J, et al. Evaluation of a mHealth Data Quality Intervention to Improve Documentation of Pregnancy Outcomes by Health Surveillance Assistants in Malawi: A Cluster Randomized Trial. Krukowski R, editor. PLoS One [Internet]. 2016 Jan 5;11(1):e0145238. Available from: https://dx.plos.org/10.1371/journal.pone.0145238
11. Vallières F, McAuliffe E, Van Bavel B, J. Wall P, Trye A. There's No App for That: Assessing the Impact of mHealth on the Supervision, Motivation, Engagement, and Satisfaction of Community Health Workers in Sierra Leone. Ann Glob Heal [Internet]. 2017 Mar 8;82(5):936. Available from: https://annalsofglobalhealth.org/article/10.1016/j.aogh.2016.07.002/
12. Ward VC, Raheel H, Weng Y, Mehta KM, Dutt P, Mitra R, et al. Impact Of Mhealth Interventions for Reproductive, Maternal, Newborn and Child Health and Nutrition at Scale: Bbc Media Action and The Ananya Program in Bihar, India. J Glob Health. 2020;10(2):1–13.
13. Carmichael SL, Mehta K, Srikantiah S, Mahapatra T, Chaudhuri I, Balakrishnan R, et al. Use of mobile technology by frontline health workers to promote reproductive, maternal, newborn and child health and nutrition: A cluster randomized controlled trial in Bihar, India. J Glob Health. 2019;9(2).

For peer review only - http://bmjopen.bmj.com/site/about/guidelines.xhtml

14. MoHFW India. National Rural Health Mission (2005-2012): Mission Document [Internet]. New Delhi; 2005. Available from: http://mohfw.nic.in/NRHM/Documents/NRHM Mission Document.pdf
15. MoHFW India. ASHA Guidelines. New Delhi: National Rural Health Mission, Ministry of Health and Family Welfare, Government of India; 2006.
16. Jhalani M. Education qualification 10th standard for ASHA selection 22.08.2012. New Delhi: Ministry of Health and Family Welfare, Government of India; 2012.
17. MoHFW. Ninth Common Review Mission Report 2015. Ministry of Health and Family Welfare, Government of India; 2015.
18. Mohan D, Shah N, Scott K, Ummer O, Chamberlain S, Lefevre AE. ASHA competency assessment in Madhya Pradesh. forthcoming.
19. DeRenzi B, Wacksman J, Dell N, Lee S, Lesh N, Borriello G, et al. Closing the Feedback Loop: A 12-month Evaluation of ASTA, a Self-Tracking Application for ASHAs. Proc Eighth Int Conf Inf Commun Technol Dev [Internet]. 2016;22:1--22:10. Available from: http://doi.acm.org/10.1145/2909609.2909652
20. NHSRC. ASHA: Which way forward? Evaluation of the ASHA Programme. Mission NRH, editor. New Delhi: National Health Systems Resource Centre; 2011.
21. Gopalan SS, Durairaj V. Addressing maternal healthcare through demand side financial incentives: experience of Janani Suraksha Yojana program in India. BMC Health Serv Res [Internet]. 2012;12(1):319. Available from: http://www.scopus.com/inward/record.url?eid=2-s2.0-84867562946&partnerID=tZOtx3y1
22. Malini S, Tripathi RM, Khattar P, Nair KS, Tekhre YL, Dhar N, et al. A Rapid Appraisal on Fuctioning of janani Suraksha Yojana in South Orrisa. Heal Poluplation. 2008;31(2):126–31.
23. Sidney K, Tolhurst R, Jehan K, Diwan V, De Costa A. "The money is important but all women anyway go to hospital for childbirth nowadays" - a qualitative exploration of why women participate in a conditional cash transfer program to promote institutional deliveries in Madhya Pradesh, India. BMC Pregnancy Childbirth [Internet]. 2016;16(1):47. Available from: http://www.pubmedcentral.nih.gov/articlerender.fcgi?artid=4779242&tool=pmcentrez&rendertype=abstract
24. Padda P, Devgun S, Gupta V, Chaudhari S, Singh G. Role of ASHA in improvement of maternal health status in northern India: An urban rural comparison. Indian J Community Heal. 2013;25(4):465–71.
25. Mony P, Raju M. Evaluation of ASHA programme in Karnataka under the National Rural Health Mission. BMC Proc [Internet]. 2012;6(Suppl 5):P12. Available from: http://www.biomedcentral.com/1753-6561/6/S5/P12
26. Wagner AL, Bettampadi D, Porth JM, Boulton ML. The impact of increased use of ASHAs on rural immunization coverage in India. 17th Int Congr Infect Dis [Poster] [Internet]. 2016;45:209–10. Available from: http://dx.doi.org/10.1016/j.ijid.2016.02.478
27. Awasthi S, Nichter M, Verma T, Srivastava NM, Agarwal M, Singh JV, et al. Revisiting community case management of childhood pneumonia: Perceptions of caregivers and grass root health providers in Uttar Pradesh and Bihar, Northern India. PLoS One. 2015;10(4):1–19.
28. Bhandari N, Mazumder S, Taneja S, Sommerfelt H, Strand TA, Group ES. Effect of

For peer review only - http://bmjopen.bmj.com/site/about/guidelines.xhtml

implementation of Integrated Management of Neonatal and Childhood Illness (IMNCI) programme on neonatal and infant mortality: cluster randomised controlled trial. BMJ. 2012;1634(March):1–13.
29. Mohan P, Kishore B, Singh S, Bahl R, Puri A, Kumar R. Assessment of implementation of integrated management of neonatal and childhood illness in India. J Heal Popul Nutr [Internet]. 2011;29(6):629–38. Available from: http://www.ncbi.nlm.nih.gov/pubmed/22283037
30. Mishra A. The role of the Accredited Social Health Activists in effective health care delivery: evidence from a study in South Orissa. BMC Proc [Internet]. 2012;6(Suppl 1):1–2. Available from: http://search.ebscohost.com/login.aspx?direct=true&db=aph&AN=71341372&site=ehost-live
31. Shrivastava SR, Shrivastava PS. Evaluation of trained Accredited Social Health Activist (ASHA) workers regarding their knowledge, attitude and practices about child health. Rural Remote Health. 2012;12(4):1–7.
32. Sinha LN, Kaur P, Gupta R, Dalpath S, Goyal V, Murhekar M. Newborn care practices and home-based postnatal newborn care programme - Mewat, Haryana, India, 2013. West Pacific Surveill response J WPSAR [Internet]. 2014;5(3):Russmann, W., Konig, U., Schlimbach, K., Pawlowska. Available from: http://www.scopus.com/inward/record.url?eid=2-s2.0-84965191350&partnerID=tZOtx3y1
33. Sharma R, Webster P, Bhattacharyya S. Factors affecting the performance of community health workers in India: A multi-stakeholder perspective. Glob Health Action. 2014;7(1):1–8.
34. Ramadurg U, Vidler M, Charanthimath U, Katageri G, Bellad M, Mallapur A, et al. Community health worker knowledge and management of pre-eclampsia in rural Karnataka State, India. Reprod Health [Internet]. 2016;13(Suppl 2):113. Available from: http://www.ncbi.nlm.nih.gov/pubmed/27719678
35. Bansal SC, Nimbalkar SM, Shah NA, Shrivastav RS, Phatak AG. Evaluation of knowledge and skills of home based newborn care among Accredited Social Health Activists (ASHA). Indian Pediatr. 2016;53(8):689–91.
36. Kori S, Bhatia M, Mishra A. A cross-sectional assessment of knowledge of ASHA workers. J Krishna Inst Med Sci Univ [Internet]. 2015;4(4):57–63. Available from: http://www.jkimsu.com/jkimsu-vol4no4/JKIMSU, Vol. 4, No. 4, Oct-Dec 2015 Page 57-63.pdf%5Cnhttp://ovidsp.ovid.com/ovidweb.cgi?T=JS&PAGE=reference&D=emed13&NEWS=N&AN=2015482227
37. UIA India. State/UT wise Aadhaar Saturation. 2018.
38. MoHFW India. National Family Health Survey (NFHS-4), India, 2015-16: Rajasthan [Internet]. Mumbai; 2017. Available from: http://www.rchiips.org/nfhs
39. NITI Aayog. Maternal Mortality Ratio (MMR) (per 100000 live births), Rajasthan, 2014-2016 [Internet]. Maternal Mortality Ratio: India, EAG& Assam, Southern States and Other States (per 100000 live births). 2019 [cited 2019 Dec 20]. Available from: https://niti.gov.in/content/maternal-mortality-ratio-mmr-100000-live-births
40. Lefevre AE, Mohan D, Scott K, Chamberlain S. Swipe out: The digital skill gap between men and women in rural Madhya Pradesh. to be Submitt.
41. Bashingwa JJH, Shah N, Mohan D, Scott K, Chamberlain S, Mulder N, et al. Examining

For peer review only

For peer review only - http://bmjopen.bmj.com/site/about/guidelines.xhtml

Reach and Exposure of a Mobile Phone-Based Training Program for frontline health workers (ASHAs) in India. BMJ Glob Heal.
42. Amoakoh-Coleman M, Borgstein ABJ, Sondaal SFV, Grobbee DE, Miltenburg AS, Verwijs M, et al. Effectiveness of mHealth interventions targeting health care workers to improve pregnancy outcomes in low- and middle-income countries: A systematic review. J Med Internet Res. 2016;18(8):1–18.
43. P. L, R. R, G.C.Y. L, G. D, Ž. C, J. C. Influences on the Implementation of Mobile Learning for Medical and Nursing Education: Qualitative Systematic Review by the Digital Health Education Collaboration. J Med Internet Res [Internet]. 2019;21(2):e12895. Available from: http://www.embase.com/search/results?subaction=viewrecord&from=export&id=L626580093%0Ahttp://dx.doi.org/10.2196/12895
44. Tudor Car L, Riboli-Sasco EF, Marcano Belisario JS, Nikolaou CK, Majeed A, Zary N, et al. Mobile learning for delivering health professional education. Cochrane Database Syst Rev. 2018;2018(6).
45. Scott K, Shinde A, Ummer O, Yadav S, Sharma M, Purty N, et al. Freedom within a cage: How patriarchal gender norms limit women's access to and use of mobile phones in rural central India. BMJ Glob Heal.
46. MoHFW. Fifth Common Review Mission Report 2011. National Rural Health Mission, Ministry of Health and Family Welfare, Government of India; 2011.
47. Ved R, Scott K. Counseling Is a Relationship Not Just a Skill: Re-conceptualizing Health Behavior Change Communication by India's Accredited Social Health Activists. Glob Heal Sci Pract. 2020;8(3).
48. Perry HB, Zulliger R, Scott K, Javadi D, Gergen J, Shelley K, et al. Case Studies of Large-Scale Community Health Worker Programs: Examples from Afghanistan, Bangladesh, Brazil, Ethiopia, India, Indonesia, Iran, Nepal, Niger, Pakistan, Rwanda, Zambia, and Zimbabwe [Internet]. Washington, DC; 2017. Available from: http://www.mcsprogram.org/wp-content/uploads/2017/01/CHW-CaseStudies-Globes.pdf

For peer review only

For peer review only - http://bmjopen.bmj.com/site/about/guidelines.xhtml

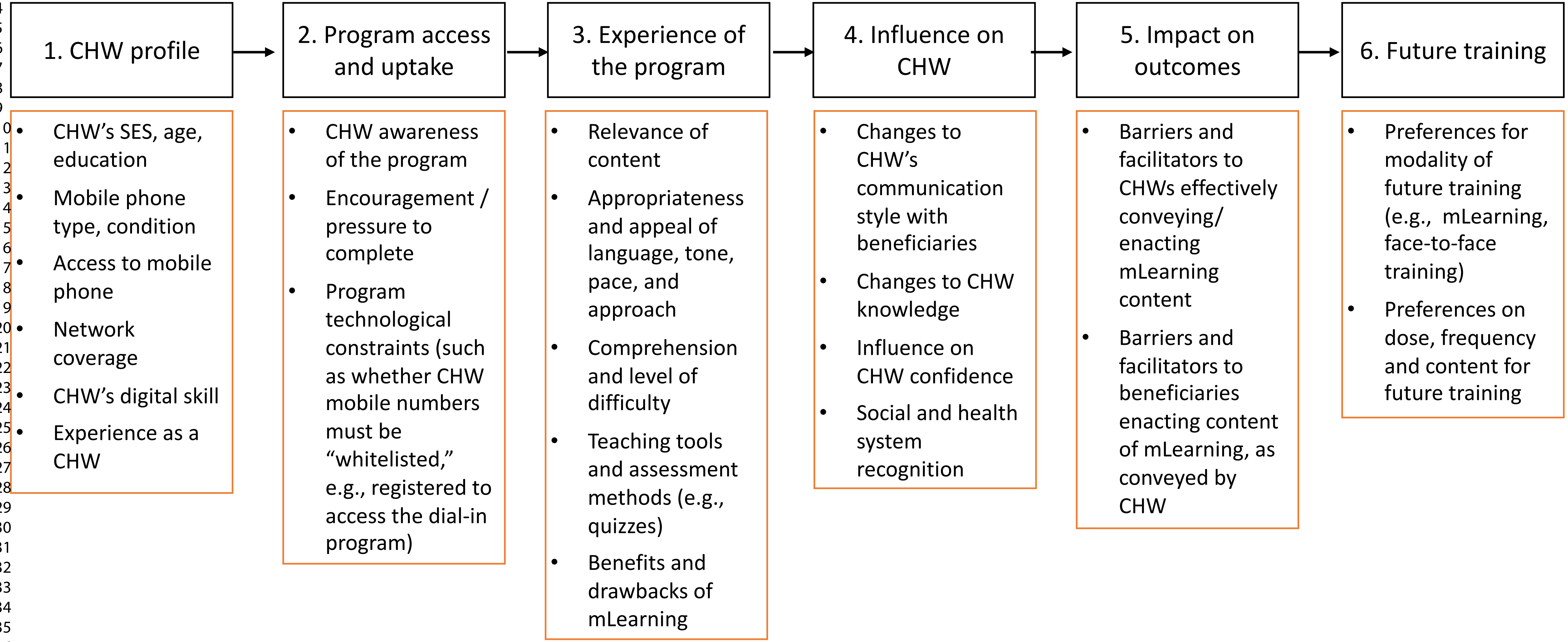
1. CHW profile
2. Program access and uptake
3. Experience of the program
4. Influence on CHW
5. Impact on outcomes
6. Future training
CHW's SES, age, education
Mobile phone type, condition
Access to mobile phone
Network coverage
CHW's digital skill
Experience as a CHW
CHW awareness of the program
Encouragement / pressure to complete
Program technological constraints (such as whether CHW mobile numbers must be "whitelisted," e.g., registered to access the dial-in program)
Relevance of content
Appropriateness and appeal of language, tone, pace, and approach
Comprehension and level of difficulty
Teaching tools and assessment methods (e.g., quizzes)
Benefits and drawbacks of mLearning
Changes to CHW's communication style with beneficiaries
Changes to CHW knowledge
Influence on CHW confidence
Social and health system recognition
Barriers and facilitators to CHWs effectively conveying/ enacting mLearning content
Barriers and facilitators to beneficiaries enacting content of mLearning, as conveyed by CHW
Preferences for modality of future training (e.g., mLearning, face-to-face training)
Preferences on dose, frequency and content for future training

Capacity to access mLearning program	mLearning program uptake	Engagement with mLearning program	Influence on CHW	Impact on outcomes	CHW desire for future training
• Mobile phone access<br>• Network coverage<br>• Digital literacy	• Learning about the program<br>• Encouragement/pressure to complete the program<br>• Support to access the program (e.g., white list number)	• Appropriateness and appeal of the tone, pace and approach<br>• Comprehension and level of difficulty<br>• Use of teaching aids (e.g., quizzes)<br>• Social support to complete<br>• Relevance of content<br>• Benefits and drawbacks of mLearning compared to face-to-face learning	• Changes to CHW's communication style with beneficiaries<br>• Changes to CHW knowledge<br>• Influence on CHW confidence<br>• Social and health system recognition	• Barriers and facilitators to CHWs effectively conveying mLearning content<br>• Barriers and facilitators to beneficiaries enacting content of mLearning, as conveyed by CHW	• Preference for mLearning compared to face-to-face learning<br>• Dose, frequency, content preferences for future mLearning

COREQ (COnsolidated criteria for REporting Qualitative research) Checklist

A checklist of items that should be included in reports of qualitative research. You must report the page number in your manuscript where you consider each of the items listed in this checklist. If you have not included this information, either revise your manuscript accordingly before submitting or note N/A.

Topic	Item No.	Guide Questions/Description	Reported on Page No.
**Domain 1: Research team and reflexivity**			
Personal characteristics			
Interviewer/facilitator	1	Which author/s conducted the interview or focus group?	
Credentials	2	What were the researcher's credentials? E.g. PhD, MD	
Occupation	3	What was their occupation at the time of the study?	
Gender	4	Was the researcher male or female?	
Experience and training	5	What experience or training did the researcher have?	
Relationship with participants			
Relationship established	6	Was a relationship established prior to study commencement?	
Participant knowledge of the interviewer	7	What did the participants know about the researcher? e.g. personal goals, reasons for doing the research	
Interviewer characteristics	8	What characteristics were reported about the inter viewer/facilitator? e.g. Bias, assumptions, reasons and interests in the research topic	
**Domain 2: Study design**			
Theoretical framework			
Methodological orientation and Theory	9	What methodological orientation was stated to underpin the study? e.g. grounded theory, discourse analysis, ethnography, phenomenology, content analysis	
Participant selection			
Sampling	10	How were participants selected? e.g. purposive, convenience, consecutive, snowball	
Method of approach	11	How were participants approached? e.g. face-to-face, telephone, mail, email	
Sample size	12	How many participants were in the study?	
Non-participation	13	How many people refused to participate or dropped out? Reasons?	
Setting			
Setting of data collection	14	Where was the data collected? e.g. home, clinic, workplace	
Presence of non-participants	15	Was anyone else present besides the participants and researchers?	
Description of sample	16	What are the important characteristics of the sample? e.g. demographic data, date	
Data collection			
Interview guide	17	Were questions, prompts, guides provided by the authors? Was it pilot tested?	
Repeat interviews	18	Were repeat inter views carried out? If yes, how many?	
Audio/visual recording	19	Did the research use audio or visual recording to collect the data?	
Field notes	20	Were field notes made during and/or after the inter view or focus group?	
Duration	21	What was the duration of the inter views or focus group?	
Data saturation	22	Was data saturation discussed?	
Transcripts returned	23	Were transcripts returned to participants for comment and/or	

For peer review only

Topic	Item No.	Guide Questions/Description	Reported on Page No.
		correction?	
**Domain 3: analysis and findings**			
Data analysis			
Number of data coders	24	How many data coders coded the data?	
Description of the coding tree	25	Did authors provide a description of the coding tree?	
Derivation of themes	26	Were themes identified in advance or derived from the data?	
Software	27	What software, if applicable, was used to manage the data?	
Participant checking	28	Did participants provide feedback on the findings?	
Reporting			
Quotations presented	29	Were participant quotations presented to illustrate the themes/findings? Was each quotation identified? e.g. participant number	
Data and findings consistent	30	Was there consistency between the data presented and the findings?	
Clarity of major themes	31	Were major themes clearly presented in the findings?	
Clarity of minor themes	32	Is there a description of diverse cases or discussion of minor themes?	

Developed from: Tong A, Sainsbury P, Craig J. Consolidated criteria for reporting qualitative research (COREQ): a 32-item checklist for interviews and focus groups. *International Journal for Quality in Health Care*. 2007. Volume 19, Number 6: pp. 349 – 357

Once you have completed this checklist, please save a copy and upload it as part of your submission. DO NOT include this checklist as part of the main manuscript document. It must be uploaded as a separate file.

For peer review only

BMJ Open

“[We] learned how to speak with love”: a qualitative exploration of Accredited Social Health Activist (ASHA) community health worker experiences of the Mobile Academy refresher training in Rajasthan, India

Journal:	*BMJ Open*
Manuscript ID	bmjopen-2021-050363.R1
Article Type:	Original research
Date Submitted by the Author:	29-Dec-2021
Complete List of Authors:	Scott, Kerry; Johns Hopkins University Bloomberg School of Public Health, International Health<br>Ummer, Osama; Oxford Policy Management; BBC Media Action<br>Chamberlain, Sara; BBC Media Action<br>Sharma, Manjula; unaffiliated , Independent researcher<br>Gharai, Dipanwita; Oxford Policy Management<br>Mishra, Bibha; Oxford Policy Management<br>Choudhury, Namrata; Jawaharlal Nehru University<br>LeFevre, Amnesty; Johns Hopkins University Bloomberg School of Public Health; University of Cape Town
**Primary Subject Heading**:	Health services research
Secondary Subject Heading:	Global health, Health policy, Medical education and training, Qualitative research
Keywords:	Health policy < HEALTH SERVICES ADMINISTRATION & MANAGEMENT, MEDICAL EDUCATION & TRAINING, Human resource management < HEALTH SERVICES ADMINISTRATION & MANAGEMENT, International health services < HEALTH SERVICES ADMINISTRATION & MANAGEMENT

SCHOLARONE™
Manuscripts

I, the Submitting Author has the right to grant and does grant on behalf of all authors of the Work (as defined in the below author licence), an exclusive licence and/or a non-exclusive licence for contributions from authors who are: i) UK Crown employees; ii) where BMJ has agreed a CC-BY licence shall apply, and/or iii) in accordance with the terms applicable for US Federal Government officers or employees acting as part of their official duties; on a worldwide, perpetual, irrevocable, royalty-free basis to BMJ Publishing Group Ltd ("BMJ") its licensees and where the relevant Journal is co-owned by BMJ to the co-owners of the Journal, to publish the Work in this journal and any other BMJ products and to exploit all rights, as set out in our licence.

The Submitting Author accepts and understands that any supply made under these terms is made by BMJ to the Submitting Author unless you are acting as an employee on behalf of your employer or a postgraduate student of an affiliated institution which is paying any applicable article publishing charge ("APC") for Open Access articles. Where the Submitting Author wishes to make the Work available on an Open Access basis (and intends to pay the relevant APC), the terms of reuse of such Open Access shall be governed by a Creative Commons licence – details of these licences and which Creative Commons licence will apply to this Work are set out in our licence referred to above.

Other than as permitted in any relevant BMJ Author's Self Archiving Policies, I confirm this Work has not been accepted for publication elsewhere, is not being considered for publication elsewhere and does not duplicate material already published. I confirm all authors consent to publication of this Work and authorise the granting of this licence.

For peer review only

"[We] learned how to speak with love": a qualitative exploration of Accredited Social Health Activist (ASHA) community health worker experiences of the Mobile Academy refresher training in Rajasthan, India

Authors: Kerry Scott[1], Osama Ummer[2], Sara Chamberlain[3], Manjula Sharma[4], Dipanwita Gharai[2], Bibha Mishra[2], Namrata Choudhury[5], and Amnesty LeFevre[1,6]

[1] Department of International Health, Johns Hopkins Bloomberg School of Public Health, 615 N. Wolfe St, Baltimore, Maryland, USA
[2] Oxford Policy Management-Delhi, 4/6 First Floor, Siri Fort Institutional Area, New Delhi, Delhi 110049, India
[3] BBC Media Action, India, Innov8 Old Fort Saket District Mall, Saket District Centre, Sector 6, Pushp Vihar, New Delhi, Delhi 110017, India
[4] Independent researcher, New Delhi, India
[5] Centre for the Study of Law and Governance, Jawaharlal Nehru University
[6] School of Public Health and Family Medicine, University of Cape Town, Cape Town, South Africa

Corresponding author: Kerry Scott, 615 N. Wolfe St, Baltimore, MD, 21205, kerry.e.scott@gmail.com, 647-675-7956.

Words in main text: 5835

For peer review only

Abstract

Introduction

Mobile Academy is a mobile-based training course for India's Accredited Social Health Activist (ASHA) community health workers. The course, which ASHAs access by dialling a number from their phones, totals four hours of audio content. It consists of 11 chapters, each with their own quiz, and provides a cumulative pass or fail score at the end. This qualitative study of Mobile Academy explores how the program was accessed and experienced by community health workers, and how they perceive it to have influenced their work.

Methods

We conducted in-depth interviews (n=25) and focus group discussions (n=5) with ASHAs and other health system actors. Open ended questions explored ASHA perspectives on Mobile Academy, the course's perceived influence on ASHAs, and preferences for future training

programs. After applying *a priori* codes to the transcripts, we identified emergent themes and grouped them according to our CHW mLearning framework.

Results

ASHAs reported enjoying Mobile Academy, specifically praising its friendly tone and the ability to repeat content. They, and higher level health systems actors, conceived it to primarily be a test not a training. ASHAs reported that they found the quizzes easy but generally did not consider the course overly simplistic. ASHAs considered Mobile Academy's content to be a useful knowledge refresher but said its primary benefit was in modelling a positive communications approach, which inspired them to adopt a kinder, more "loving" communication style when speaking to beneficiaries. ASHAs and health system actors wanted follow-on mLearning courses that would continue to compliment but not replace face-to-face training.

Conclusion

This mLearning program for community health workers in India was well received by ASHAs across a wide range of education levels and experience. Dial-in audio training has the potential to not only reinforce topical knowledge but also showcase positive ways to communicate.

Keywords

mobile phones; mLearning; training and supervision; community health workers; frontline health workers; ASHAs; mHealth; mLearning; digital literacy; India

For peer review only

For peer review only - http://bmjopen.bmj.com/site/about/guidelines.xhtml

Strengths and limitations of this study

- The user perspectives on Mobile Academy presented here are vital to understanding the potential of an mHealth training strategy that requires minimal supervisor input and can be used by CHWs with low or no literacy, limited digital skills, and no smartphone or Internet access – common realities for community health workers in low resource settings.
- Our purposive sampling strategy using backend Mobile Academy data enables us to present insight into a variety of user experiences, including perspectives from typical users and a sub-set of atypically highly educated and digitally savvy ASHAs.
- We are limited to presenting the experiences of ASHAs in the Indian state of Rajasthan; given that Mobile Academy has been scaled up across 13 states, additional localized research is required.
- We present ASHA perspectives on how the program influenced their knowledge and communication with beneficiaries; evaluating these assertions was beyond the scope of our research.
- The time lag between when ASHAs took Mobile Academy (between 2016 and early 2018) and when we interviewed them about it (late 2018) may have reduced the emotional intensity of their responses or introduced inaccurate recollections of program components.

Introduction

Community health workers (CHWs), a type of frontline health worker (FLHWs), have been widely recognised as an integral part of primary health care teams, particularly in low and middle income countries (LMICs).[1,2] Globally, CHWs perform a range of health system functions to improve the acceptability and reach of health services, including counselling and health education, encouraging care-seeking and treatment adherence, and improving the reach of health care to marginalized communities.[3] However, CHW performance is often hindered by a range of programme and health system limitations, including insufficient training and supervision.[3,4]

With the rapid rise in mobile phone access and network connectivity in LMICs, the use of mobile technology for health (mHealth) has flourished.[5] mHealth strategies have been identified as one mechanism to improve CHW training and support, although evidence of effectiveness is mixed, partially because of the diversity in interventions.[6–8] Digital tools developed specifically for FLHW training range from simple didactic programs such as informational text messages to complex eLearning courses.[9] WhatsApp groups bolstered supportive supervision for Kenyan CHWs[10] and motivational SMS messages improved the documentation of pregnancies by CHWs in Malawi.[11] However, a human resources

For peer review only - http://bmjopen.bmj.com/site/about/guidelines.xhtml

management tool called MOTECH did not improve perceived supervision, self-reported motivation, work engagement and job satisfaction among Sierra Leonean CHWs.[12] Research from Bihar, India found that Mobile Kunji, a job aid for Accredited Social Health Worker (ASHA) CHWs and *anganwadi* nutrition workers, increased time spent with beneficiaries, improved beneficiary perceptions of health worker trustworthiness, and increased some positive health behaviours such as exclusive breastfeeding.[13] Continuum of Care Services, a job aid for India's FLHWs, increased the frequency of FLHW-beneficiary interactions and of FLHW coordination of home visits. It also improved job confidence and increased beneficiary practice of skin-to-skin care, immediate breastfeeding and appropriate complementary feeding.[14] It did not improve FLHW supervision or other health-related behaviours among beneficiaries. User perceptions and acceptability of digital training have broadly been found to be positive. FLHWs have found digital training programs easy to use and the content easy to understand[15–17] and relevant.[18] However, few studies have conducted indepth examination of FLHW perspectives on mLearning, including how FLHWs with different profiles engage with the technology and content, motivation and hesitation around a new form of training, appropriateness of content, perceived impact, and how they compare them to face-to-face options.

This paper explores user and health system actor perceptions of Mobile Academy, a mobile phone-based interactive voice response (IVR) training program, which was developed for India's FLHWs, including ASHAs. IVR programs deliver audio recordings to any type of phone and allows users to provide feedback by pressing a number key on the keypad. Mobile Academy, developed by BBC Media Action in India, provides ASHAs with refresher training on reproductive, maternal, neonatal and child health and seeks to help them better communicate with community members. The program consists of 44 short (2.5 minute) pre-recorded audio lessons (for a total of 240 minutes). The ASHAs answer 4 multiple choice quiz questions at the end of each of the 11 chapters (for a total of 44 questions) to reinforce the messages and build their confidence, including on using phones for learning. ASHAs dial in to a toll-free number to access the course and can listen to as much content as they like before ending the call. Bookmarking technology allows them to return to the lesson where they left off the next-time they call in. At the end of the course, ASHAs who score 50% or higher receive an SMS notifying them of their completion with a unique number; they can show this number to the primary health centre administration to receive a completion certificate. In half of the 13 states where Mobile Academy has been launched, 50% to 75% of all ASHAs have successfully completed the course. But in other states, uptake is below 15%.

While the program has been scaled up by the Government of India and completed by over 180,000 ASHAs to date, knowledge about user perceptions of Mobile Academy, its influence on interpersonal communication, and ASHA reflections on future mLearning opportunities remains limited. This paper presents the results of a qualitative study exploring these issues in Rajasthan, a state in northern India.

Methods

For peer review only - http://bmjopen.bmj.com/site/about/guidelines.xhtml

ASHA program and training

ASHAs are female CHWs who facilitate antenatal care, institutional deliveries, and immunizations, provide home-based newborn care, promote family planning, hygiene, and nutrition, and convene village health events.[19,20] Current recruitment standards (released in 2012) dictate that ASHAs are required to have completed 10 years of formal education; ASHAs recruited in the first eight years of the program (2005 – 2012) required only 8 years' education and educational requirements can be "relaxed" in marginalized regions.[21] While some ASHAs have attained education beyond high school, non-literate ASHAs are not uncommon in many states[22]; 17% of ASHAs surveyed in Madhya Pradesh were unable to read a full sentence [23], and 30% of ASHAs surveyed in Uttar Pradesh were low literate.[24] ASHAs have been found to average over 20 work hours a week,[25] and receive a small fixed monthly honorarium as well as performance based remuneration with total monthly income ranging from Rs 900 to Rs 4250 (USD$14 to $65) depending on state-level top up payments and ASHA activity. ASHAs are also compensated Rs 200 (USD$3) for attending face to face trainings. ASHAs have long asserted that they are underpaid.[26,27]

State governments aim to provide ASHAs with 23 days initial training and 10 days supplementary training, as well as monthly supportive supervision through the government primary healthcare system.[28] Early evaluations identified curriculum inadequacies and low quality training delivery as major issues facing the program.[28,29] Over time, trainings have sought to re-orient towards a competency based approach, with efforts to balance health knowledge, clinical skills and communication and counseling ability.[30] While the program has been credited with bolstering antenatal care, increasing institutional deliveries[31–34] and childhood immunization[35,36] and improving community case management of childhood illness,[37–39] studies have consistently identified gaps in ASHA knowledge and skills including on childhood illness and dangers signs in pregnancy. [37,39–46] Beyond gaps in knowledge and skills, shortcomings in ASHAs' interpersonal and counselling skills have also been identified.[30,47–49] Their communicative and counselling efficacy is influenced by their training on communication and counselling,[48–50] as well as personal characteristics (e.g., the ASHA's confidence, empowerment, education), power hierarchies (e.g., caste, class, religion, gender), other identity and relational factors (e.g., geographic proximity, political affiliations, marital status, number of children, and family relationship histories),[43,51,52] and the health system more broadly (e.g., the extent to which ASHAs are able to linked to clinics that provide high quality health care).[53,54]

Study setting

Rajasthan is a majority Hindi-speaking state in Northern India with a population of 78 million.[55] While 85% of men are literate, literacy among women is only 57%.[56] The maternal mortality ratio is 199 per 100,000 live births[57] and the rural under-five mortality rate is 54.4 per 1000 live births.[56] Only 39% of women receive the recommended four antenatal care checkups, but 84% of deliveries take place in health facilities.[56] As of January 2018, there were approximately 44,900 ASHAs in the state's rural areas, which is 88% of the targeted recruitment and amounts to 1 ASHA per 1147 rural people.

For peer review only - http://bmjopen.bmj.com/site/about/guidelines.xhtml

Sampling

We received permission to conduct this qualitative study in three districts (Ajmer, Sikar and Pali) of Rajasthan, selected by government authorities. Data collection took place in October and November 2018. ASHAs were selected purposively, using data generated by Mobile Academy's technical system and guidance from block-level ASHA facilitators. We interviewed ASHAs who had completed Mobile Academy when it was officially launched (2016, early 2017), as well as ASHAs who completed Mobile Academy more recently (late 2017, 2018). This decision was made to explore possible differences between early and late completers of Mobile Academy. We also hypothesized that late completers would be more likely to recall specific details of the course, since it would be fresher in their minds. We sought ASHAs with varied Mobile Academy engagement profiles. We considered the number of minutes an ASHA was engaged in the course (while most ASHAs in Rajasthan completed the course in approximately 240 minutes, we sought 11 ASHAs who took 270 minutes or more), their scores (while most ASHAs in Rajasthan had a score in the 40s out of a maximum of 44 points, we sampled 15 ASHAs with scores less than 40/44), and ASHAs who had repeated the course (we sampled four ASHAs who went through the course twice). Our FGDs included a mix of ASHAs by Mobile Academy completion data, nature of Mobile Academy engagement (quiz scores etc.) and education level. We also interviewed district and state level actors (Table 1).

Table 1. Mobile Academy respondent sample

Respondent	Male	Female	Total
In-depth interviews			
• ASHA	0	25	25
• ANM	0	6	6
• ASHA supervisor	3	3	6
• Block ASHA coordinator	2	1	3
• District actor	3	0	3
• State actor	1	1	2
Total	9	36	45
Focus group discussions			
• ASHAs (5 to 6 participants)	0	5	5

Recruitment, data collection and ethics

This study was approved by the institutional ethics review boards at Sigma, Delhi, India (10041/IRB/D/17-18) and JHU, Baltimore, USA (00008360). Interviews and FGDs were conducted by four female qualitative researchers (authors MS, DG, BM and NC), supported by a male research manager (OU) and female research coordinator (KS). All researchers were trained over a one-week period, which included pilot testing the detailed ASHA interview guide, and had master's level social science education or higher. They approached the respondents first by phone to explain the study, identify themselves as working for a Delhi-based research company, explain that they had governmental approval to conduct this study, and ask if they could meet face to face to learn more and, if they agreed, to participate. The interviews took about an hour and were conducted in ASHA homes and in

For peer review only - http://bmjopen.bmj.com/site/about/guidelines.xhtml

other stakeholder's offices and health facilities; the focus group discussions took just over an hour and were conducted in empty school buildings, clinics and courtyards. ASHA family members were often around the home during the ASHA interviews but did not actively listen to or engage with the interviews. Only the researchers and participants were present during the other data collection. The study information and informed consent was read to each potential participant and then summarized in conversational language to ensure comprehension. All respondents provided informed oral consent. Two people who we approached for the study refused: one ASHA who had a family emergency and one state level actor citing lack of interest. All respondents who agreed to participate in the study also allowed audio recording. The research domains explored are presented in box 1.

Box 1. Research domains explored

In-depth interviews

- Relationship with mobile technology (respondents' access to and use of mobile phones);
- Perception of Mobile Academy (voice, pace, content, relevance);
- Mobile Academy uptake (what motivated ASHAs to complete the course, how did they hear about it, did they encounter technological or social barriers to accessing it);
- Mobile Academy quizzes (difficulty, how respondent felt about them, use of the phone keypad to answer questions);
- Mobile Academy completion certificate (process for receiving certificate, importance of certificate);
- Extent to which Mobile Academy is a refresher training versus providing new content;
- Mobile Academy's influence on ASHA-beneficiary interaction and relationships (listening to Mobile Academy with others, telling others about Mobile Academy, drawing from Mobile Academy to help in communication with beneficiaries; the influence of Mobile Academy on ASHAs' confidence, respect and motivation);
- ASHA face-to-face trainings (content, benefits, drawbacks, relationships among ASHAs and between ASHAs and trainers, link to Mobile Academy);
- Desire for further training, especially through mobile technology (content, modality, dose)

Focus group discussions

- General perspectives of Mobile Academy;
- Challenges that ASHAs navigate in trying to promote birth preparedness and family planning;
- Whether Mobile Academy had any influence on how ASHAs spoke to people about these issues;
- ASHA perspectives on future mLearning.

Analysis

Analysis began with daily debrief meetings, wherein the field team (MS, DG, BM, NC, OU and KS) drew from interview notes to discuss emerging themes and adjust elements of the

For peer review only - http://bmjopen.bmj.com/site/about/guidelines.xhtml

guide and sampling based on saturation and emergent sub-topics without the study domains. After data collection, all audio files were transcribed and translated into English and uploaded into Dedoose, a qualitative data management software. Guided by the principles of thematic analysis, KS and OU developed and applied a coding framework with nine code clusters which echoed the topics explored in the interviews and FGDs. The clusters consisted of between three and 11 codes which were primarily drawn from *a priori* areas of interest.

After coding, we generated code reports that enabled us to read all text tagged by the same code. While reviewing these reports we were mindful of the respondent profiles and ASHA-ANM-ASHA facilitator clusters to identify any patterns or trends that existed. We present our findings thematically, grouped under the five components of our framework for understanding CHW engagement with mobile learning courses (Fig 1).

[Figure 1. Framework for understanding CHW engagement with mobile learning courses]

Patient and Public Involvement

The research was shaped by ASHA and other health system actor priorities, experiences and preferences through iterative probing and flexibility within our research domains. Results were disseminated to Government of India stakeholders and actors involved in developing Mobile Academy.

Findings

1. CHW profile

Mobile Academy was considered relevant and appropriate by ASHAs across a wide range of demographic and engagement profiles

While the ASHAs in our five focus groups were selected based only on having completed Mobile Academy and being geographically close to one another, the 25 ASHAs selected for interviews respondent sample was non-typical in several ways (table 2). We sought the rare ASHAs who completed Mobile Academy late (2018 or late 2017), in a state where 93% of ASHAs completed Mobile Academy in 2016 and early 2017. Our late completers (n=12) turned out to be newly recruited ASHAs who were also more educated than early completers (n=13).[21] This selection skewed our respondent sample towards ASHAs with higher education, which is not representative of ASHAs in the state. Whereas only 2 (8%) of the ASHAs interviewed in our study had eight or fewer years of education, over 70% of Rajasthan's ASHAs did.[28] However, our five focus group discussions were composed primarily of "typical" ASHAs (recruited into the ASHA program in the mid-2000s, fewer than 10 years of education, completed Mobile Academy early, and achieved high scores), which enabled us to round out the range of perspectives captured in this research.

For peer review only - http://bmjopen.bmj.com/site/about/guidelines.xhtml

Table 2. ASHA respondents interviewed in this study compared to ASHAs overall in Rajasthan state

Parameter	Our IDI sample n (%) (n=25)	Rajasthan N (%) (n= see notes)
Education		
<=8	2 (8%)	72%*
9 – 10	7 (28%)	23%*
11 – 12	11 (44%)	3%*
Graduate	5 (20%)	1%*
Mobile Academy		
Early completion (2016, early 2017)	13 (52%)	41,164 (93%)**
Late completion (late 2017, 2018)	12 (48%)	1,440 (3%)**
Average quiz score (out of 44 points)	31.6	42.6**
Failed (<22/44)	6 (24%)	91 (0.2%)**
Average duration (minutes)***	291.2	255.0[#]
ASHAs who repeated the course	3 (12%)	533 (13%)[#]

*From *ASHA Which Way Forward* (2011) based on a sample of 200 ASHAs
** All ASHAs who completed Mobile Academy in Rajasthan (n=44,545)
*** Of first course. If an ASHA repeated the course, this value would approximately double, but we are not considering this second attempt here, since we report repeats in a separate row.
# Only ASHAs who completed Mobile Academy in Sikar, Pali and Ajmer Districts

We did not identify a qualitative difference between early and late completers in terms of their recall of content or their interpretation of Mobile Academy's appropriateness and ease. Both groups reported that the course was a knowledge assessment and a refresher training, which covered the same content as their core face-to-face trainings. They reported that the course was relevant and appropriate, and easy to understand. ASHAs across the spectrum of education described the quizzes as easy and enjoyable.

We also sought ASHAs for interviews who had lower scores and who went through Mobile Academy more than once. We included six ASHAs who failed (24% of our sample) when only 0.2% of ASHAs in Rajasthan failed Mobile Academy. Surprisingly, the six ASHAs who failed told us that they are unaware that they had performed poorly – despite the fact that the course informs users when they have failed – and, like the ASHAs with high scores, described the course as "easy". All three ASHAs who repeated Mobile Academy (ASHA_11, ASHA_12 and ASHA_24) told us that they did so because their supervisors told them that their first attempt was not properly logged in the system, and that they had to complete the course again. This explanation is likely a miscommunication or misunderstanding because our records show that each attempt was logged.

ASHAs in our sample had universal mobile phone access and variable digital literacy

All the ASHAs in our sample reported having their own phones, which they carried with them when they went out and could use without asking permission. In many households, the ASHA's phone was also used by other family members. Almost half the ASHAs in our sample reported that they used a smart phone, which indicates a more digitally-enabled group of ASHAs compared to the norm; in Madhya Pradesh a recent survey found that only 11% of ASHAs had a smartphone.[58] Even among our savvy qualitative sample, internet access was variable since few ASHAs bought mobile internet data regularly. (Internet access

For peer review only

had no bearing on Mobile Academy access because Mobile Academy is a dial-in mobile phone service.) ASHAs explained that the government-issued SIM for ASHA-related activities allowed them to speak to other ASHAs and ASHA facilitators and make calls to beneficiaries; however in some areas this government SIM did not work because of network issues or late payment of monthly bills by the government. ASHAs kept a second, personal, SIM, which they sometimes used for ASHA work as well.

Some ASHAs reported receiving calls from government actors, including their ANM and ASHA facilitator, as well as health system actors in district headquarters, the state capital and Delhi. These calls were to inform them about trainings and meetings, ask ASHAs to report data such as the number of pregnancies, or, in one case, to ask the ASHA to follow up with specific high risk patients. Others had not received any ASHA-work related phone calls. Over half of the ASHAs in this qualitative sample were on WhatsApp, compared to 12% in a representative sample of ASHAs in Madhya Pradesh.[58] Our ASHAs reported using WhatsApp to share reports and information with and among ASHAs, however access to WhatsApp was irregular and many ASHAs explained that their husbands were actually in the ASHA WhatsApp groups but reported information from the WhatsApp groups back to them.

All ASHAs were confident that they could receive calls from beneficiaries about emergencies, such as women in labor, and could make outgoing calls to the ambulance and to families, often to notify them about vaccinations and health checkups. Beyond receiving and making calls, most ASHAs self reported that they could open and read SMS and WhatsApp messages but several disclosed that they got stuck sometimes doing these activities. Our research team did not test these capacities. Most said that they were not able to write and send messages by SMS or in WhatsApp, even among those who completed education beyond 12th standard: “Neither do I know how to send SMS nor do I send. It is not required.” (ASHA_08, 44/44, graduate). Several ASHAs described needing help from family members to operate their phones, including saving contacts and opening and reading messages.

2. Program access and uptake

Mobile Academy was widely understood to be primarily an examination

ASHAs and higher-level health system stakeholders described Mobile Academy as an ASHA knowledge test or examination rather than a refresher course. Relatedly, several higher level stakeholders (DIST_01, BLCK_01, BLCK_02) considered Mobile Academy to be an assessment of the ASHAs and were concerned that Mobile Academy could be completed by other people or that ASHAs could cheat by sharing the answers to the quizzes.

ASHAs completed Mobile Academy because of pressure from above, but also expressed desire to gain knowledge

ASHAs described pressure to complete Mobile Academy “*aage se* [from above].” They repeatedly reported being told by their supervisors that completing the course was *zaroori* [necessary/essential] and were also aware that the ANM and ASHA supervisor were under pressure from district level authorities to have all their ASHAs finish Mobile Academy.

For peer review only

> There was pressure, it's important to do it… we were getting messages, phone calls, again and again, that this is important for you to do. (FGD_ASHA_03)

A few ASHAs described being worried that they would lose their positions if they did not complete Mobile Academy or if they did not pass. One ASHA also reported being told that ASHA pay would increase upon completing Mobile Academy, which was not the case (FGD_ASHA_03). ASHAs were not paid for completing Mobile Academy, and when asked about payment they tended to say it was not necessary. Instead, they framed the course was a means to remain in their positions and excel at their work. It was only when discussing future mLearning opportunities (section 5) that several ASHAs mentioned that they ought to be paid for these activities.

In addition to being required to take the course, ASHAs described being motivated to completed Mobile Academy by their desire to gain more knowledge, as well as general curiosity, an interest in receiving the certificate and a desire to show higher level health system actors that they are knowledgeable.

> We get to learn, that it the reason. There is a will to learn, as an ASHA - for ourselves and for the villagers, like our family, like our village (ASHA_15, 43/44, 12th)

Mobile Academy uptake among our sample did not follow planned dose and frequency

While ASHAs could call into Mobile Academy and proceed through the course whenever they had time over weeks or even months, the ASHAs we spoke to reported completing it rapidly in just one or two days. National level data on Mobile Academy engagement found that 36% of ASHAs completed the course within three days, and 12% within the first 24 hours.[59] Our data can offer insight into what might have driven this sizable minority of ASHAs to proceed so rapidly. Some of the ASHAs in our sample reported not knowing that the course could be completed in parts and some were unsure how much time could elapse between starting and completing the course. Others worried about not being able to get through to the Mobile Academy line if they called back later. Many also emphasised that they were under pressure to complete the course quickly.

3. Experience of Mobile Academy

Initial fear replaced by happiness; ASHAs praised the pace and tone, valued repetition

When ASHAs first heard that they were to complete a training by mobile phone, they described feeling nervous. They did not know what it would entail or were concerned about having to provide answers to quiz questions. However, they quickly relaxed and enjoyed the course because of the warm and friendly tone, option to repeat content, and the fact that the quiz questions covered familiar topics and were easy.

For peer review only - http://bmjopen.bmj.com/site/about/guidelines.xhtml

> At first, I was very scared that I would give the wrong answer. Then when I finished it, I felt very good. (FGD_ASHA_05)

> I thought I do not know what she will ask and what she will not ask. This was the fear. But after listening I was not afraid. (ASHA_23, 38/44, 10th)

ASHAs expressed very positive overall opinions of the course. The character voices were frequently described as loving, warm and calm: "Sweetness was there in the voice" (ASHA_24, 44/44, 12th, early adopter). The Hindi language speech was easy to understand for the ASHAs, although they generally spoke Marwadi, a Rajasthani dialect close to Hindi.

> Her voice was good and sweet. […] She spoke exactly spoke like us. (ASHA_29, 35/44, 12th)

> We could trust her well. She was talking in such a way that it was nobody else but someone of her own, apna hi hai [like she knows us]. (ASHA_15, 43/44. 12th)

> They explained with a lot of love, very slowly. And asked [questions] calmly. (FGD_ASHA_03)

ASHAs felt the pace was appropriate: "neither it was fast nor slow; everything was explained in a proper manner" (ASHA_29) and greatly valued being able to repeat content. ASHAs emphasized that the repetitive content and ability to re-listen to lessons was a major strength of the program: "they were repeating some of the topics two to three times and then we were able to remember that nicely" (ASHA_31, 21/44, 8th).

Quizzes increased ASHA confidence

As designed, the ASHAs experienced quizzes to be easy but enjoyable. Although described as "easy" by all, ASHAs still said they had to pay attention properly in order to complete them.

> ASHA1: [I] got 100 out of 100. I felt like, if more like these are happening, we will do it.
> ASHA2: We had a lot of fun, lot of fun
> ASHA3: That we cleared the paper [passed the test]! If we get the next paper [test], we will clear that too.
> ASHA2: We passed it easily. We didn't have to listen to anyone scolding us.
> [all laugh]
> ASHA4: I felt like I passed a competition
> ASHA3: I was so happy to get good marks (FGD_ASHA_03)

> Absolutely we have to keep our mind on one point. Whatever they tell we have to find out the answer from that immediately. We must not delay. (ASHA_24, 44/44, 12th)

As mentioned above, those with low quiz scores were unaware that there was any issue with their score and described the quizzes as "easy". None reported receiving feedback that they had failed, despite the fact that the course reports back to users whenever they select an incorrect quiz answer and if they have failed at the end, suggesting that ASHAs forgot, did not realize, or did not want to admit to our research team that they had struggled with the quiz.

Mobile Academy is often a social activity

Despite the individual dial-in design, many ASHAs reported involving other people. Some (ASHA_31, FGD_ASHA_05) reported proceeding through Mobile Academy with a group of other ASHAs during group training sessions. Some played the content for colleagues, such as anganwadi workers and ASHA supervisors, to show them what the course was about or to seek assistance. Others played Mobile Academy on speakerphone for their children or in-laws to explain why they were on the phone for a long duration and to share the content.

Mobile Academy a complement to face-to-face training

ASHAs greatly valued that mobile phone-based training allowed them to complete the course from home, when they had spare time. This home-based model avoided the difficulties of making childcare arrangements and leaving behind domestic responsibilities inherent to face-to-face training or meetings.

> Otherwise [for face-to-face training] we have to leave the family and children and go. We have tension in that case. But here we can sit back at home and do the training. We can do it whenever we get time. (ASHA_03, 44/44, 10th)

Furthermore, ASHAs in remote areas valued that mLearning did not require the time and discomfort of travelling long distances to reach training centres. Two ASHAs (ASHA_11, ASHA_18) also noted that they could focus better alone at home compared to group settings where multiple people were speaking at once.

> We can listen to it peacefully. There, [face-to-face training] ten people are sitting. Who is saying what? Here we can listen comfortably. (ASHA_11, 42/44, 8th)

Several ASHAs contrasted the kind and encouraging approach of Mobile Academy with the punitive and demoralizing approach to training and supervision that they experienced face-to-face. ASHAs reported being frightened of supervisors and trainers, explaining that no ASHAs speak because "we feel a bit scared", "if we are wrong he will scold us" (FGD_ASHA_03); "they scold us badly" (FGD_ASHA_05). However, other ASHAs found face-to-face trainers to be supportive and noted that in-person training offered the opportunity to ask questions whenever they did not understand; to learn techniques and practices by seeing them demonstrated; to connect with other ASHAs ("all the ASHAs get together, they talk and gossip" (FGD_ASHA_05)), trainers and facilitators; and to get out of the house.

> ASHA1: Sister, it's like, in training face to face we will get to learn more. […] In Mobile Academy, we can only listen. In the other one, we will get to learn –
> ASHA2: The way/technique
> ASHA1: Practical
> ASHA3: They show us
> ASHA2: In face to face training, madam, if we ask questions we also get answers. We can ask also, what, why is this. In that [Mobile Academy], we can't even ask. We can just say yes or no. (FGD_ASHA_04)

Most ASHAs explained that both modalities of training had benefits and drawbacks, and thus hoped to receive further face-to-face training, supplemented with mLearning.

For peer review only - http://bmjopen.bmj.com/site/about/guidelines.xhtml

4. Mobile Academy's influence on ASHAs

Mobile Academy showcased a positive communication style that ASHAs can apply when speaking to beneficiaries

When asked about Mobile Academy's influence, ASHA most commonly explained that the course changed how they communicated with beneficiaries. They explained that they modelled the tone used by the voice actors in Mobile Academy to speak to beneficiaries, introducing a more loving and calm approach to their communication with women:

> [We] learned how to speak with *prem* [love] (laughs) [...] After doing Mobile Academy, we've made some changes in the way we talk. [...] This only: explain once. If they don't understand then explain again, but explain with love. Don't get angry, that won't work there. (FGD_ASHA_03)

They spoke of internalizing the importance of taking time to talk to beneficiaries, not rushing, and repeating content in a caring manner.

Mobile Academy increased ASHA confidence and reinforced knowledge

ASHAs reported feeling more confident and knowledgeable after completing Mobile Academy. As noted above, Mobile Academy was designed as a refresher training that covered the same topics as face-to-face training; ASHAs reported experiencing it in this way and said that the refresher content enabled them to retain some specific knowledge.

> If they tell us again, it makes a difference. [...] We are able to understand [now] that the training happened for Mobile Academy. The course refreshes things. [Face-to-face] trainings happened here 8 to 10 years ago." (FGD_ASHA_03)

While ASHAs reported that they did not tell beneficiaries that they completed Mobile Academy and did not listen to Mobile Academy with beneficiaries, they drew from their training, including Mobile Academy, to answer beneficiary questions.

> Earlier we did not have courage to talk in front of others. Now we have courage. If we did not know anything, what would we tell others? If we have a little bit of knowledge only then we can tell others. If anybody asks anything, we can answer them as well. (ASHA_15, 43/44, 12th)

While ASHAs were to receive Mobile Academy completion certificates, many did not. ASHAs who had not received a certificate did not articulate personal disappointment or concern about community perception. However, some worried that they lacked proof to show health system stakeholders that they completed the course.

5. Future training

ASHAs and higher-level health system actors were very positive about integrating future mLearning alongside face-to-face training to reinforce, refresh, or deepen understanding.

For peer review only - http://bmjopen.bmj.com/site/about/guidelines.xhtml

ASHAs wanted future Mobile Academy content to cover newborn care, family planning, nutrition, non-communicable disease (diabetes, hypertension and cancer), adolescent health, village health committees, tuberculosis, malaria, leprosy, and cataracts. They said that future mobile training could consist of up to six hours of content, spread in half hour increments over several weeks or months. When asked directly about incentives to complete future trainings most but not all ASHAs requested payment. One noted that they are paid for face-to-face trainings and said that mLearning ought not to be different: "We get money for every training. So if we get in this one, it is good." (ASHA_11) However, several said that no money was needed, explaining that the knowledge itself is desirable ("Because we have to learn something, so why should they pay money?" ASHA_29) and instead requested other incentives such as certificates or the opportunity for promotion.

Discussion

This qualitative exploration of Mobile Academy found strong positive perceptions of the course among ASHAs and other health system actors. ASHAs found Mobile Academy material relevant and appropriate, were driven to take the course because of a desire to learn and pressure from supervisors. They appreciated its warm and friendly tone and their ability to repeat content. ASHAs valued the course for refreshing and deepening their knowledge as well as showcasing a positive communication style that they could apply to interactions with beneficiaries. Respondents were enthusiastic about future mLearning, although they noted that face-to-face training could not be replaced with mobile phone-based programs.

In addition to these outcomes, several surprising aspects of ASHA engagement with the course emerged. First, although Mobile Academy was designed for ASHAs with eight years of education, ASHAs with 12 or more years of education also said that they valued the course. As ASHA educational requirements and mobile phone savvy increase, research examining whether some ASHAs can handle more complex material, alternative teaching styles, and the more sophisticated digital engagement used elsewhere will be valuable.[60–62] Nonetheless, given the persistence of low literacy and limited digital skills among women in India[63] including ASHAs[23,24,64], ongoing investment in audio based trainings that can be accessed from any phone, rather than textual or smartphone based programming, remains critical.

Second, although Mobile Academy was designed to be a training program with simple quiz questions that served only to motivate and engage ASHAs, the quiz component became the focus. Further research is needed to understand the range of effective ways to engage interactive feedback as motivators, teaching tools, and systems for assessment and accreditation. Third, the ASHAs in our sample tended to complete the course rapidly, in one or two long sessions, rather than spreading the content over many weeks. Nationally, a smaller portion (36%) took a rapid approach.[59] The ASHAs in our qualitative study explained that they would have liked to proceed through the course more slowly, but took the rapid approach because of pressure from supervisors and concern that they might be unable to access the course again later. This finding, as well as the outsized focus on the quizzes,

For peer review only - http://bmjopen.bmj.com/site/about/guidelines.xhtml

suggests that supervisors and FLHWs require steady communication about the intent and engagement options for mLearning programs.

Fourth, ASHAs and health system actors understood Mobile Academy as an internal accreditation rather than an achievement that could bolster community trust. Other studies from South Asia have found that women's engagement with mobile phones enables "modern" aspirations for education and socially mobility but requires reputational risk management.[63,65] The ways in which ASHA use of digital technology affects their social identities and relationships warrants further research.

ASHAs reported that Mobile Academy's lasting impact on them was both informational and affective – ASHAs spontaneously and frequently focused on the "loving" communication style and their intention to incorporate this into their interactions with beneficiaries. A few ASHAs contrasted the kind tone in Mobile Academy to a more intimidating teaching style used at some face-to-face trainings. Power hierarchies in the Indian health system (e.g., by education, caste, professional status, gender, urban-rural residence) can be reproduced in trainer-ASHA dynamics and in ASHA-beneficiary interactions, ultimately limiting communication and trust throughout the system.[54,66] The capacity for digital trainings to improve communication within this hierarchical space require further exploration: to what extent does Mobile Academy subvert hierarchies versus engage with them in a productive manner? Can face-to-face trainers learn from Mobile Academy's tone and approach? And what is the actual impact of this "loving" tone on ASHA-beneficiary interactions? Ultimately, it is important to note that strengthening ASHA-beneficiary relationships will require ongoing ASHA capacity building (of which mLearning can play a role), community engagement, and quality improvements in the government health services themselves.[67]

This study was limited to the experiences of ASHAs in the Indian state of Rajasthan. Given that Mobile Academy has been scaled up across 13 states, additional localized research would be beneficial. Another limitation of this study is that we only present ASHA and supervisor perspectives on how Mobile Academy influenced ASHA knowledge and communication with beneficiaries. Evaluating these assertions was beyond the scope of our research but would be very beneficial. Finally, the time lag between when ASHAs took Mobile Academy (between 2016 and early 2018) and when we interviewed them about it (late 2018) may have reduced the emotional intensity of their responses or introduced inaccurate recollections of program components.

Conclusion

While mLearning programs have been found capable of improving aspects of FLHW knowledge, patient engagement, and supervision, they tend to require smartphone technology and user digital skills that remain out of reach to many of India's rural FLHWs.[38,39] Many also rely on trainer inputs such as remote moderation and feedback[10], that are challenging add-ons in overstretched systems such as India's where vacancies throughout the supervisory hierarchy remain an issue.[68] Mobile Academy required only a

For peer review only

basic phone and the ability to use a keypad to access its pre-recorded content and interactive quiz questions. It was well received by the ASHA FLHWs in our sample, which included a wide range of education levels and experience, including many ASHAs with more education and better digital access than the norm. Policymakers can note Mobile Academy's high acceptability among users, as well as its potential to not only reinforce knowledge but also encourage positive beneficiary-FLHW communication style. These findings position the Mobile Academy model as a viable mLearning option for many LMIC contexts to reinforce ongoing face-to-face training.

Acknowledgements

The authors are grateful to the ASHAs and other health system actors who generously provided their time and insights to make this research possible. This work was made possible by the Bill and Melinda Gates Foundation. We thank Diva Dhar, Suhel Bidani, Rahul Mullick, Dr. Suneeta Krishnan, Dr. Neeta Goel and Dr. Priya Nanda for believing in us and giving us this opportunity. We additionally thank Vinit Pattnaik at OPM and Erica Crawford at Johns Hopkins for their support to the financial management of our work.

Data sharing

Data for this study consist of qualitative interview and focus group discussion transcripts. Uploading all transcripts for open availability would compromise our ability to fully mask participant details. However, we are happy to share anonymized portions of these transcripts upon reasonable request.

Competing interests

All authors have completed the Unified Competing Interest form (available on request from the corresponding author) and declare that the research reported was funded by the Bill and Melinda Gates Foundation. SC is employed by BBC Media Action, one of the entities supporting program implementation for Mobile Academy. The authors do not have other relationships and are not engaged in activities that could appear to have influenced the submitted work.

Contributor statement

KS, SC , OU and AEL conceptualized and designed the study. MS, DG, BM and NC conducted the data collection and preliminary data analysis through daily analytic debriefs. KS and OU led and managed the data collection and analysis, including the coding and thematic synthesis. KS drafted the manuscript and revised it based on critical and substantive input from SC, OU, AEL, MS, BM and NC. All authors agree to be accountable for all aspects of the work related to accuracy and integrity.

For peer review only

Funding statement

This work was supported by the Bill and Melinda Gates Foundation grant number OPP1179252.

Works Cited

1. WHO. The World Health Report 2008. Primary Health Care - Now more than ever [Internet]. Vol. 26, The World Health Report. Geneva, Switzerland; 2008. Available from: http://www.who.int/whr/2008/whr08_en.pdf
2. WHO. Global Strategy on Human Resources for Health: Workforce 2030 [Internet]. Synthesis Paper of the Thematic Working Groups. Geneva; 2016. Available from: http://www.who.int/hrh/resources/pub_globstrathrh-2030/en/
3. Scott K, Beckham S, Gross M, Pariyo G, Rao K, Cometto G, et al. What do we know about community-based health programs? A systematic review of existing reviews on community health workers and their integration with health systems. Hum Resour Health. 2018;1–17.
4. Cometto G, Ford N, Pfaffman-Zambruni J, Akl EA, Lehmann U, McPake B, et al. Health policy and system support to optimise community health worker programmes: an abridged WHO guideline. Lancet Glob Heal [Internet]. 2018;6(12):e1397–404. Available from: http://dx.doi.org/10.1016/S2214-109X(18)30482-0
5. Marcolino MS, Oliveira JAQ, D'Agostino M, Ribeiro AL, Alkmim MBM, Novillo-Ortiz D. The Impact of mHealth Interventions: Systematic Review of Systematic Reviews. JMIR mHealth uHealth [Internet]. 2018 Jan 17;6(1):e23–e23. Available from: https://www.ncbi.nlm.nih.gov/pubmed/29343463
6. Agarwal S, Perry HB, Long LA, Labrique AB. Evidence on feasibility and effective use of mHealth strategies by frontline health workers in developing countries: Systematic review. Trop Med Int Heal. 2015;20(8):1003–14.
7. O'Donovan J, Bersin A, O'Donovan C. The effectiveness of mobile health (mHealth) technologies to train healthcare professionals in developing countries: a review of the literature. BMJ Innov [Internet]. 2015 Jan;1(1):33–6. Available from: http://innovations.bmj.com/lookup/doi/10.1136/bmjinnov-2014-000013
8. O'Donovan J, O'Donovan C, Kuhn I, Sachs SE, Winters N. Ongoing training of community health workers in low-income and middle-income countries: a systematic scoping review of the literature. BMJ Open [Internet]. 2018 Apr 1;8(4):e021467. Available from: http://bmjopen.bmj.com/content/8/4/e021467.abstract
9. Schoeman F. Digital Tools for Training Frontline Health Workers in Low- and Middle-Income Countries: A Systematic Review. Univeristy of Cape Town; 2019.
10. Henry JV, Winters N, Lakati A, Oliver M, Geniets A, Mbae SM, et al. Enhancing the Supervision of Community Health Workers With WhatsApp Mobile Messaging: Qualitative Findings From 2 Low-Resource Settings in Kenya. Glob Heal Sci Pract [Internet]. 2016 Jun 20;4(2):311–25. Available from: http://www.ghspjournal.org/lookup/doi/10.9745/GHSP-D-15-00386
11. Joos O, Silva R, Amouzou A, Moulton LH, Perin J, Bryce J, et al. Evaluation of a mHealth Data Quality Intervention to Improve Documentation of Pregnancy Outcomes by Health Surveillance Assistants in Malawi: A Cluster Randomized Trial. Krukowski R, editor. PLoS One [Internet]. 2016 Jan 5;11(1):e0145238. Available from: https://dx.plos.org/10.1371/journal.pone.0145238

For peer review only - http://bmjopen.bmj.com/site/about/guidelines.xhtml

12. Vallières F, McAuliffe E, Van Bavel B, J. Wall P, Trye A. There's No App for That: Assessing the Impact of mHealth on the Supervision, Motivation, Engagement, and Satisfaction of Community Health Workers in Sierra Leone. Ann Glob Heal [Internet]. 2017 Mar 8;82(5):936. Available from: https://annalsofglobalhealth.org/article/10.1016/j.aogh.2016.07.002/
13. Ward VC, Raheel H, Weng Y, Mehta KM, Dutt P, Mitra R, et al. Impact Of Mhealth Interventions for Reproductive, Maternal, Newborn and Child Health and Nutrition at Scale: Bbc Media Action and The Ananya Program in Bihar, India. J Glob Health. 2020;10(2):1–13.
14. Carmichael SL, Mehta K, Srikantiah S, Mahapatra T, Chaudhuri I, Balakrishnan R, et al. Use of mobile technology by frontline health workers to promote reproductive, maternal, newborn and child health and nutrition: A cluster randomized controlled trial in Bihar, India. J Glob Health. 2019;9(2).
15. Diedhiou A, Gilroy KE, Cox CM, Duncan L, Koumtingue D, Pacque-Margolis S, et al. Successful mLearning pilot in Senegal: Delivering family planning refresher training using interactive voice response and SMS. Glob Heal Sci Pract. 2015;3(2):305–21.
16. Bellina L, Azzolina G, Nucatola I, Maggio A, Consiglio F, Ingrassia R. m-phone impact on practical training: role of m-phone as part of the educative method for training local workers of rural areas of developing countries. Health Technol. 2014;4:289–94.
17. Limaye R, Deka S, Ahmed N, Mwaikambo L. Designing eLearning courses to meet the digital literacy needs of healthcare workers in lower- and middle-income 71 countries: Experiences from the Knowledge for Health Project. Knowl Manag E-Learning. 2015;7(4):601–15.
18. Pimmer C, Mbvubdula F. One message, many voices: Mobile audio counselling in health education. J Health Care Poor Underserved. 2018;29(1):463–80.
19. MoHFW India. National Rural Health Mission (2005-2012): Mission Document [Internet]. New Delhi; 2005. Available from: http://mohfw.nic.in/NRHM/Documents/NRHM Mission Document.pdf
20. MoHFW India. ASHA Guidelines. New Delhi: National Rural Health Mission, Ministry of Health and Family Welfare, Government of India; 2006.
21. Jhalani M. Education qualification 10th standard for ASHA selection 22.08.2012. New Delhi: Ministry of Health and Family Welfare, Government of India; 2012.
22. MoHFW. Ninth Common Review Mission Report 2015. Ministry of Health and Family Welfare, Government of India; 2015.
23. Mohan D, Shah N, Scott K, Ummer O, Chamberlain S, Lefevre AE. ASHA competency assessment in Madhya Pradesh. forthcoming.
24. DeRenzi B, Wacksman J, Dell N, Lee S, Lesh N, Borriello G, et al. Closing the Feedback Loop: A 12-month Evaluation of ASTA, a Self-Tracking Application for ASHAs. Proc Eighth Int Conf Inf Commun Technol Dev [Internet]. 2016;22:1--22:10. Available from: http://doi.acm.org/10.1145/2909609.2909652
25. MoHFW. Update on the ASHA Programme, January 2017 [Internet]. New Delhi, India; Available from: http://nhsrcindia.org/sites/default/files/Update on ASHA Programme- January-2017.pdf
26. Bhatia K. Community health worker programs in India: a rights-based review. Perspect Public Health. 2014;134(5):276–82.
27. Bhatia K. Performance based incentives of the ASHA Scheme: Stakeholders' Perspectives. Econ Polit Wkly. 2014;49(22):145.

For peer review only - http://bmjopen.bmj.com/site/about/guidelines.xhtml

28. NHSRC. ASHA: Which way forward? Evaluation of the ASHA Programme. Mission NRH, editor. New Delhi: National Health Systems Resource Centre; 2011.
29. Nirupam B, Dholakia RH. Improving the performance of Accredited Social Health Activists in India. Working Paper No. 1. Mumbai: Columbia Global Centers | South Asia, Columbia University; 2011.
30. Shrivastava A, Srivastava A. Measuring communication competence and effectiveness of ASHAs (accredited social health activist) in their leadership role at rural settings of Uttar Pradesh (India). Leadersh Heal Serv [Internet]. 2016;29(1):69–81. Available from: http://www.emeraldinsight.com/doi/10.1108/LHS-12-2014-0079
31. Gopalan SS, Durairaj V. Addressing maternal healthcare through demand side financial incentives: experience of Janani Suraksha Yojana program in India. BMC Health Serv Res [Internet]. 2012;12(1):319. Available from: http://www.scopus.com/inward/record.url?eid=2-s2.0-84867562946&partnerID=tZOtx3y1
32. Malini S, Tripathi RM, Khattar P, Nair KS, Tekhre YL, Dhar N, et al. A Rapid Appraisal on Fuctioning of janani Suraksha Yojana in South Orrisa. Heal Poluplation. 2008;31(2):126–31.
33. Sidney K, Tolhurst R, Jehan K, Diwan V, De Costa A. "The money is important but all women anyway go to hospital for childbirth nowadays" - a qualitative exploration of why women participate in a conditional cash transfer program to promote institutional deliveries in Madhya Pradesh, India. BMC Pregnancy Childbirth [Internet]. 2016;16(1):47. Available from: http://www.pubmedcentral.nih.gov/articlerender.fcgi?artid=4779242&tool=pmcentrez&rendertype=abstract
34. Padda P, Devgun S, Gupta V, Chaudhari S, Singh G. Role of ASHA in improvement of maternal health status in northern India: An urban rural comparison. Indian J Community Heal. 2013;25(4):465–71.
35. Mony P, Raju M. Evaluation of ASHA programme in Karnataka under the National Rural Health Mission. BMC Proc [Internet]. 2012;6(Suppl 5):P12. Available from: http://www.biomedcentral.com/1753-6561/6/S5/P12
36. Wagner AL, Bettampadi D, Porth JM, Boulton ML. The impact of increased use of ASHAs on rural immunization coverage in India. 17th Int Congr Infect Dis [Poster] [Internet]. 2016;45:209–10. Available from: http://dx.doi.org/10.1016/j.ijid.2016.02.478
37. Awasthi S, Nichter M, Verma T, Srivastava NM, Agarwal M, Singh JV, et al. Revisiting community case management of childhood pneumonia: Perceptions of caregivers and grass root health providers in Uttar Pradesh and Bihar, Northern India. PLoS One. 2015;10(4):1–19.
38. Bhandari N, Mazumder S, Taneja S, Sommerfelt H, Strand TA, Group ES. Effect of implementation of Integrated Management of Neonatal and Childhood Illness (IMNCI) programme on neonatal and infant mortality: cluster randomised controlled trial. BMJ. 2012;1634(March):1–13.
39. Mohan P, Kishore B, Singh S, Bahl R, Puri A, Kumar R. Assessment of implementation of integrated management of neonatal and childhood illness in India. J Heal Popul Nutr [Internet]. 2011;29(6):629–38. Available from: http://www.ncbi.nlm.nih.gov/pubmed/22283037
40. Mishra A. The role of the Accredited Social Health Activists in effective health care

For peer review only - http://bmjopen.bmj.com/site/about/guidelines.xhtml

delivery: evidence from a study in South Orissa. BMC Proc [Internet]. 2012;6(Suppl 1):1–2. Available from: http://search.ebscohost.com/login.aspx?direct=true&db=aph&AN=71341372&site=ehost-live

41. Shrivastava SR, Shrivastava PS. Evaluation of trained Accredited Social Health Activist (ASHA) workers regarding their knowledge, attitude and practices about child health. Rural Remote Health. 2012;12(4):1–7.

42. Sinha LN, Kaur P, Gupta R, Dalpath S, Goyal V, Murhekar M. Newborn care practices and home-based postnatal newborn care programme - Mewat, Haryana, India, 2013. West Pacific Surveill response J WPSAR [Internet]. 2014;5(3):Russmann, W., Konig, U., Schlimbach, K., Pawlowska. Available from: http://www.scopus.com/inward/record.url?eid=2-s2.0-84965191350&partnerID=tZOtx3y1

43. Sharma R, Webster P, Bhattacharyya S. Factors affecting the performance of community health workers in India: A multi-stakeholder perspective. Glob Health Action. 2014;7(1):1–8.

44. Ramadurg U, Vidler M, Charanthimath U, Katageri G, Bellad M, Mallapur A, et al. Community health worker knowledge and management of pre-eclampsia in rural Karnataka State, India. Reprod Health [Internet]. 2016;13(Suppl 2):113. Available from: http://www.ncbi.nlm.nih.gov/pubmed/27719678

45. Bansal SC, Nimbalkar SM, Shah NA, Shrivastav RS, Phatak AG. Evaluation of knowledge and skills of home based newborn care among Accredited Social Health Activists (ASHA). Indian Pediatr. 2016;53(8):689–91.

46. Kori S, Bhatia M, Mishra A. A cross-sectional assessment of knowledge of ASHA workers. J Krishna Inst Med Sci Univ [Internet]. 2015;4(4):57–63. Available from: http://www.jkimsu.com/jkimsu-vol4no4/JKIMSU, Vol. 4, No. 4, Oct-Dec 2015 Page 57-63.pdf%5Cnhttp://ovidsp.ovid.com/ovidweb.cgi?T=JS&PAGE=reference&D=emed13&NEWS=N&AN=2015482227

47. Das E, Panwar DS, Fischer EA, Bora G, Carlough MC. Performance of accredited social health activists to provide home-based newborn care: A situational analysis. Indian Pediatr. 2014;51(2):142–4.

48. Panwar DS, Naidu V, Das E, Verma S, Khan AA. Strengthening support mechanisms for Accredited Social Health Activists in order to improve home-based newborn care in Uttar Pradesh, India. BMC Proc [Internet]. 2012;6(Suppl 5):1–2. Available from: http://search.ebscohost.com/login.aspx?direct=true&db=aph&AN=82432034&site=ehost-live

49. Goel AD, Gosain M, Amarchand R, Sharma H, Rai S, Kapoor SK, et al. Effectiveness of a Quality Improvement Program Using Difference-in-Difference Analysis for Home Based Newborn Care – Results of a Community Intervention Trial. Indian J Pediatr [Internet]. 2019;86(11):1028–35. Available from: http://www.embase.com/search/results?subaction=viewrecord&from=export&id=L2002309415%0Ahttp://dx.doi.org/10.1007/s12098-019-03012-4

50. Smittenaar P, Ramesh BM, Jain M, Blanchard J, Kemp H, Engl E, et al. Bringing Greater Precision to Interactions Between Community Health Workers and Households to Improve Maternal and Newborn Health Outcomes in India. Glob Heal Sci Pract [Internet]. 2020 Sep 30;8(3):358–71. Available from:

For peer review only - http://bmjopen.bmj.com/site/about/guidelines.xhtml

http://www.ghspjournal.org/lookup/doi/10.9745/GHSP-D-20-00027
51. Scott K, George AS, Harvey SA, Mondal S, Patel G, Ved R, et al. Beyond form and functioning: Understanding how contextual factors influence village health committees in northern India. PLoS One. 2017 Aug;12(8):e0182982.
52. Sarin E, Lunsford SS. How female community health workers navigate work challenges and why there are still gaps in their performance: A look at female community health workers in maternal and child health in two Indian districts through a reciprocal determinism framework. Hum Resour Health. 2017;15(1).
53. Ved R, Scott K. Counseling is a relationship not just a skill: Re-conceptualizing health behavior change communication by india's accredited social health activists. Glob Heal Sci Pract. 2020;8(3):332–4.
54. Scott K, Shanker S. Tying their hands? Institutional obstacles to the success of the ASHA community health worker programme in rural north India. AIDS Care. 2010;22 Suppl 2(931159996):1606–12.
55. UIA India. State/UT wise Aadhaar Saturation. 2018.
56. MoHFW India. National Family Health Survey (NFHS-4), India, 2015-16: Rajasthan [Internet]. Mumbai; 2017. Available from: http://www.rchiips.org/nfhs
57. NITI Aayog. Maternal Mortality Ratio (MMR) (per 100000 live births), Rajasthan, 2014-2016 [Internet]. Maternal Mortality Ratio: India, EAG& Assam, Southern States and Other States (per 100000 live births). 2019 [cited 2019 Dec 20]. Available from: https://niti.gov.in/content/maternal-mortality-ratio-mmr-100000-live-births
58. Lefevre AE, Mohan D, Scott K, Chamberlain S. Swipe out: The digital skill gap between men and women in rural Madhya Pradesh. to be Submitt.
59. Bashingwa JJH, Shah N, Mohan D, Scott K, Chamberlain S, Mulder N, et al. Examining Reach and Exposure of a Mobile Phone-Based Training Program for frontline health workers (ASHAs) in India. BMJ Glob Heal.
60. Amoakoh-Coleman M, Borgstein ABJ, Sondaal SFV, Grobbee DE, Miltenburg AS, Verwijs M, et al. Effectiveness of mHealth interventions targeting health care workers to improve pregnancy outcomes in low- and middle-income countries: A systematic review. J Med Internet Res. 2016;18(8):1–18.
61. P. L, R. R, G.C.Y. L, G. D, Ž. C, J. C. Influences on the Implementation of Mobile Learning for Medical and Nursing Education: Qualitative Systematic Review by the Digital Health Education Collaboration. J Med Internet Res [Internet]. 2019;21(2):e12895. Available from: http://www.embase.com/search/results?subaction=viewrecord&from=export&id=L626580093%0Ahttp://dx.doi.org/10.2196/12895
62. Tudor Car L, Riboli-Sasco EF, Marcano Belisario JS, Nikolaou CK, Majeed A, Zary N, et al. Mobile learning for delivering health professional education. Cochrane Database Syst Rev. 2018;2018(6).
63. Scott K, Shinde A, Ummer O, Yadav S, Sharma M, Purty N, et al. Freedom within a cage: How patriarchal gender norms limit women's use of mobile phones in rural central India. BMJ Glob Heal. 2021;6.
64. MoHFW. Fifth Common Review Mission Report 2011. National Rural Health Mission, Ministry of Health and Family Welfare, Government of India; 2011.
65. Huang JQ. Digital aspirations: 'wrong-number' mobile-phone relationships and experimental ethics among women entrepreneurs in rural Bangladesh. J R Anthropol Inst. 2018;24(1):107–25.

For peer review only

66. Roalkvam S. Health governance in India: citizenship as situated practice. Glob Public Health [Internet]. 2014;9(8):910–26. Available from: http://www.pubmedcentral.nih.gov/articlerender.fcgi?artid=4166913&tool=pmcentrez&rendertype=abstract
67. Ved R, Scott K. Counseling Is a Relationship Not Just a Skill: Re-conceptualizing Health Behavior Change Communication by India's Accredited Social Health Activists. Glob Heal Sci Pract. 2020;8(3).
68. Perry HB, Zulliger R, Scott K, Javadi D, Gergen J, Shelley K, et al. Case Studies of Large-Scale Community Health Worker Programs: Examples from Afghanistan, Bangladesh, Brazil, Ethiopia, India, Indonesia, Iran, Nepal, Niger, Pakistan, Rwanda, Zambia, and Zimbabwe [Internet]. Washington, DC; 2017. Available from: http://www.mcsprogram.org/wp-content/uploads/2017/01/CHW-CaseStudies-Globes.pdf

For peer review only

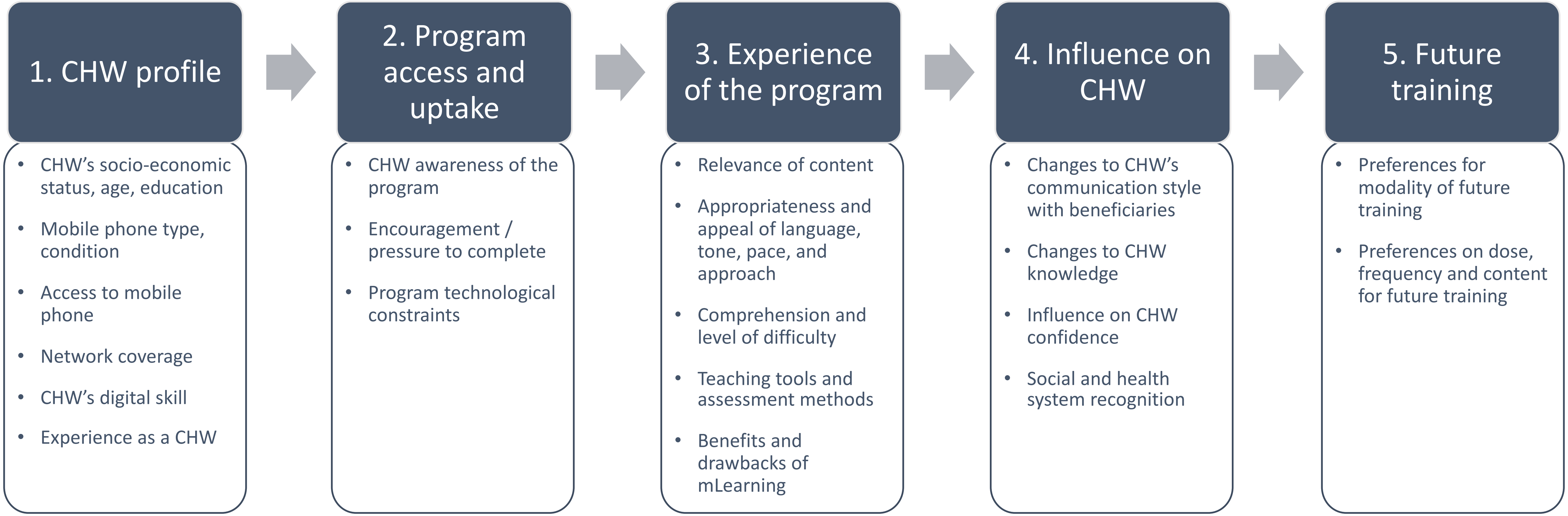
1. CHW profile
CHW's socio-economic status, age, education
Mobile phone type, condition
Access to mobile phone
Network coverage
CHW's digital skill
Experience as a CHW
2. Program access and uptake
CHW awareness of the program
Encouragement / pressure to complete
Program technological constraints
3. Experience of the program
Relevance of content
Appropriateness and appeal of language, tone, pace, and approach
Comprehension and level of difficulty
Teaching tools and assessment methods
Benefits and drawbacks of mLearning
4. Influence on CHW
Changes to CHW's communication style with beneficiaries
Changes to CHW knowledge
Influence on CHW confidence
Social and health system recognition
5. Future training
Preferences for modality of future training
Preferences on dose, frequency and content for future training

COREQ (COnsolidated criteria for REporting Qualitative research) Checklist

A checklist of items that should be included in reports of qualitative research. You must report the page number in your manuscript where you consider each of the items listed in this checklist. If you have not included this information, either revise your manuscript accordingly before submitting or note N/A.

Topic	Item No.	Guide Questions/Description	Reported on Page No.
**Domain 1: Research team and reflexivity**			
Personal characteristics			
Interviewer/facilitator	1	Which author/s conducted the interview or focus group?	
Credentials	2	What were the researcher's credentials? E.g. PhD, MD	
Occupation	3	What was their occupation at the time of the study?	
Gender	4	Was the researcher male or female?	
Experience and training	5	What experience or training did the researcher have?	
Relationship with participants			
Relationship established	6	Was a relationship established prior to study commencement?	
Participant knowledge of the interviewer	7	What did the participants know about the researcher? e.g. personal goals, reasons for doing the research	
Interviewer characteristics	8	What characteristics were reported about the inter viewer/facilitator? e.g. Bias, assumptions, reasons and interests in the research topic	
**Domain 2: Study design**			
Theoretical framework			
Methodological orientation and Theory	9	What methodological orientation was stated to underpin the study? e.g. grounded theory, discourse analysis, ethnography, phenomenology, content analysis	
Participant selection			
Sampling	10	How were participants selected? e.g. purposive, convenience, consecutive, snowball	
Method of approach	11	How were participants approached? e.g. face-to-face, telephone, mail, email	
Sample size	12	How many participants were in the study?	
Non-participation	13	How many people refused to participate or dropped out? Reasons?	
Setting			
Setting of data collection	14	Where was the data collected? e.g. home, clinic, workplace	
Presence of non-participants	15	Was anyone else present besides the participants and researchers?	
Description of sample	16	What are the important characteristics of the sample? e.g. demographic data, date	
Data collection			
Interview guide	17	Were questions, prompts, guides provided by the authors? Was it pilot tested?	
Repeat interviews	18	Were repeat inter views carried out? If yes, how many?	
Audio/visual recording	19	Did the research use audio or visual recording to collect the data?	
Field notes	20	Were field notes made during and/or after the inter view or focus group?	
Duration	21	What was the duration of the inter views or focus group?	
Data saturation	22	Was data saturation discussed?	
Transcripts returned	23	Were transcripts returned to participants for comment and/or	

For peer review only

Topic	Item No.	Guide Questions/Description	Reported on Page No.
		correction?	
**Domain 3: analysis and findings**			
Data analysis			
Number of data coders	24	How many data coders coded the data?	
Description of the coding tree	25	Did authors provide a description of the coding tree?	
Derivation of themes	26	Were themes identified in advance or derived from the data?	
Software	27	What software, if applicable, was used to manage the data?	
Participant checking	28	Did participants provide feedback on the findings?	
Reporting			
Quotations presented	29	Were participant quotations presented to illustrate the themes/findings? Was each quotation identified? e.g. participant number	
Data and findings consistent	30	Was there consistency between the data presented and the findings?	
Clarity of major themes	31	Were major themes clearly presented in the findings?	
Clarity of minor themes	32	Is there a description of diverse cases or discussion of minor themes?	

Developed from: Tong A, Sainsbury P, Craig J. Consolidated criteria for reporting qualitative research (COREQ): a 32-item checklist for interviews and focus groups. *International Journal for Quality in Health Care*. 2007. Volume 19, Number 6: pp. 349 – 357

Once you have completed this checklist, please save a copy and upload it as part of your submission. DO NOT include this checklist as part of the main manuscript document. It must be uploaded as a separate file.

For peer review only

BMJ Open

“[We] learned how to speak with love”: a qualitative exploration of Accredited Social Health Activist (ASHA) community health worker experiences of the Mobile Academy refresher training in Rajasthan, India

Journal:	*BMJ Open*
Manuscript ID	bmjopen-2021-050363.R2
Article Type:	Original research
Date Submitted by the Author:	08-Apr-2022
Complete List of Authors:	Scott, Kerry; Johns Hopkins University Bloomberg School of Public Health, International Health<br>Ummer, Osama; Oxford Policy Management; BBC Media Action<br>Chamberlain, Sara; BBC Media Action<br>Sharma, Manjula; unaffiliated , Independent researcher<br>Gharai, Dipanwita; Oxford Policy Management<br>Mishra, Bibha; Oxford Policy Management<br>Choudhury, Namrata; Jawaharlal Nehru University<br>LeFevre, Amnesty; Johns Hopkins University Bloomberg School of Public Health; University of Cape Town
**Primary Subject Heading**:	Health services research
Secondary Subject Heading:	Global health, Health policy, Medical education and training, Qualitative research
Keywords:	Health policy < HEALTH SERVICES ADMINISTRATION & MANAGEMENT, MEDICAL EDUCATION & TRAINING, Human resource management < HEALTH SERVICES ADMINISTRATION & MANAGEMENT, International health services < HEALTH SERVICES ADMINISTRATION & MANAGEMENT

SCHOLARONE™
Manuscripts

I, the Submitting Author has the right to grant and does grant on behalf of all authors of the Work (as defined in the below author licence), an exclusive licence and/or a non-exclusive licence for contributions from authors who are: i) UK Crown employees; ii) where BMJ has agreed a CC-BY licence shall apply, and/or iii) in accordance with the terms applicable for US Federal Government officers or employees acting as part of their official duties; on a worldwide, perpetual, irrevocable, royalty-free basis to BMJ Publishing Group Ltd ("BMJ") its licensees and where the relevant Journal is co-owned by BMJ to the co-owners of the Journal, to publish the Work in this journal and any other BMJ products and to exploit all rights, as set out in our licence.

The Submitting Author accepts and understands that any supply made under these terms is made by BMJ to the Submitting Author unless you are acting as an employee on behalf of your employer or a postgraduate student of an affiliated institution which is paying any applicable article publishing charge ("APC") for Open Access articles. Where the Submitting Author wishes to make the Work available on an Open Access basis (and intends to pay the relevant APC), the terms of reuse of such Open Access shall be governed by a Creative Commons licence – details of these licences and which Creative Commons licence will apply to this Work are set out in our licence referred to above.

Other than as permitted in any relevant BMJ Author's Self Archiving Policies, I confirm this Work has not been accepted for publication elsewhere, is not being considered for publication elsewhere and does not duplicate material already published. I confirm all authors consent to publication of this Work and authorise the granting of this licence.

For peer review only

"[We] learned how to speak with love": a qualitative exploration of Accredited Social Health Activist (ASHA) community health worker experiences of the Mobile Academy refresher training in Rajasthan, India

Authors: Kerry Scott[1], Osama Ummer[2], Sara Chamberlain[3], Manjula Sharma[4], Dipanwita Gharai[2], Bibha Mishra[2], Namrata Choudhury[5], and Amnesty LeFevre[1,6]

[1] Department of International Health, Johns Hopkins Bloomberg School of Public Health, 615 N. Wolfe St, Baltimore, Maryland, USA
[2] Oxford Policy Management-Delhi, 4/6 First Floor, Siri Fort Institutional Area, New Delhi, Delhi 110049, India
[3] BBC Media Action, India, Innov8 Old Fort Saket District Mall, Saket District Centre, Sector 6, Pushp Vihar, New Delhi, Delhi 110017, India
[4] Independent researcher, New Delhi, India
[5] Centre for the Study of Law and Governance, Jawaharlal Nehru University
[6] School of Public Health and Family Medicine, University of Cape Town, Cape Town, South Africa

Corresponding author: Kerry Scott, 615 N. Wolfe St, Baltimore, MD, 21205, kerry.e.scott@gmail.com, 647-675-7956.

Words in main text: 5835

Abstract

Introduction

Mobile Academy is a mobile-based training course for India's Accredited Social Health Activist (ASHA) community health workers. The course, which ASHAs access by dialling a number from their phones, totals four hours of audio content. It consists of 11 chapters, each with their own quiz, and provides a cumulative pass or fail score at the end. This qualitative study of Mobile Academy explores how the program was accessed and experienced by community health workers, and how they perceive it to have influenced their work.

Methods

We conducted in-depth interviews (n=25) and focus group discussions (n=5) with ASHAs and other health system actors. Open ended questions explored ASHA perspectives on Mobile Academy, the course's perceived influence on ASHAs, and preferences for future training

For peer review only

programs. After applying *a priori* codes to the transcripts, we identified emergent themes and grouped them according to our CHW mLearning framework.

Results

ASHAs reported enjoying Mobile Academy, specifically praising its friendly tone and the ability to repeat content. They, and higher level health systems actors, conceived it to primarily be a test not a training. ASHAs reported that they found the quizzes easy but generally did not consider the course overly simplistic. ASHAs considered Mobile Academy's content to be a useful knowledge refresher but said its primary benefit was in modelling a positive communications approach, which inspired them to adopt a kinder, more "loving" communication style when speaking to beneficiaries. ASHAs and health system actors wanted follow-on mLearning courses that would continue to compliment but not replace face-to-face training.

Conclusion

This mLearning program for community health workers in India was well received by ASHAs across a wide range of education levels and experience. Dial-in audio training has the potential to not only reinforce topical knowledge but also showcase positive ways to communicate.

Keywords

mobile phones; mLearning; training and supervision; community health workers; frontline health workers; ASHAs; mHealth; mLearning; digital literacy; India

For peer review only

Strengths and limitations of this study

- The user perspectives on Mobile Academy presented here are vital to understanding the potential of an mHealth training strategy that requires minimal supervisor input and can be used by CHWs with low or no literacy, limited digital skills, and no smartphone or Internet access – common realities for community health workers in low resource settings.
- Our purposive sampling strategy using backend Mobile Academy data enables us to present insight into a variety of user experiences, including perspectives from typical users and a sub-set of atypically highly educated and digitally savvy ASHAs.
- We are limited to presenting the experiences of ASHAs in the Indian state of Rajasthan; given that Mobile Academy has been scaled up across 13 states, additional localized research is required.
- We present ASHA perspectives on how the program influenced their knowledge and communication with beneficiaries; evaluating these assertions was beyond the scope of our research.
- The time lag between when ASHAs took Mobile Academy (between 2016 and early 2018) and when we interviewed them about it (late 2018) may have reduced the emotional intensity of their responses or introduced inaccurate recollections of program components.

Introduction

Community health workers (CHWs), a type of frontline health worker (FLHWs), have been widely recognised as an integral part of primary health care teams, particularly in low and middle income countries (LMICs).[1,2] Globally, CHWs perform a range of health system functions to improve the acceptability and reach of health services, including counselling and health education, encouraging care-seeking and treatment adherence, and improving the reach of health care to marginalized communities.[3] However, CHW performance is often hindered by a range of programme and health system limitations, including insufficient training and supervision.[3,4]

With the rapid rise in mobile phone access and network connectivity in LMICs, the use of mobile technology for health (mHealth) has flourished.[5] mHealth strategies have been identified as one mechanism to improve CHW training and support, although evidence of effectiveness is mixed, partially because of the diversity in interventions.[6–8] Digital tools developed specifically for FLHW training range from simple didactic programs such as informational text messages to complex eLearning courses.[9] WhatsApp groups bolstered supportive supervision for Kenyan CHWs[10] and motivational SMS messages improved the documentation of pregnancies by CHWs in Malawi.[11] However, a human resources

For peer review only

management tool called MOTECH did not improve perceived supervision, self-reported motivation, work engagement and job satisfaction among Sierra Leonean CHWs.[12] Research from Bihar, India found that Mobile Kunji, a job aid for Accredited Social Health Worker (ASHA) CHWs and *anganwadi* nutrition workers, increased time spent with beneficiaries, improved beneficiary perceptions of health worker trustworthiness, and increased some positive health behaviours such as exclusive breastfeeding.[13] Continuum of Care Services, a job aid for India's FLHWs, increased the frequency of FLHW-beneficiary interactions and of FLHW coordination of home visits. It also improved job confidence and increased beneficiary practice of skin-to-skin care, immediate breastfeeding and appropriate complementary feeding.[14] It did not improve FLHW supervision or other health-related behaviours among beneficiaries. User perceptions and acceptability of digital training have broadly been found to be positive. FLHWs have found digital training programs easy to use and the content easy to understand[15–17] and relevant.[18] However, few studies have conducted indepth examination of FLHW perspectives on mLearning, including how FLHWs with different profiles engage with the technology and content, motivation and hesitation around a new form of training, appropriateness of content, perceived impact, and how they compare them to face-to-face options.

This paper explores user and health system actor perceptions of Mobile Academy, a mobile phone-based interactive voice response (IVR) training program, which was developed for India's FLHWs, including ASHAs. IVR programs deliver audio recordings to any type of phone and allows users to provide feedback by pressing a number key on the keypad. Mobile Academy, developed by BBC Media Action in India, provides ASHAs with refresher training on reproductive, maternal, neonatal and child health and seeks to help them better communicate with community members. The program consists of 44 short (2.5 minute) pre-recorded audio lessons (for a total of 240 minutes). The ASHAs answer 4 multiple choice quiz questions at the end of each of the 11 chapters (for a total of 44 questions) to reinforce the messages and build their confidence, including on using phones for learning. ASHAs dial in to a toll-free number to access the course and can listen to as much content as they like before ending the call. Bookmarking technology allows them to return to the lesson where they left off the next-time they call in. At the end of the course, ASHAs who score 50% or higher receive an SMS notifying them of their completion with a unique number; they can show this number to the primary health centre administration to receive a completion certificate. In half of the 13 states where Mobile Academy has been launched, 50% to 75% of all ASHAs have successfully completed the course. But in other states, uptake is below 15%.

While the program has been scaled up by the Government of India and completed by over 180,000 ASHAs to date, knowledge about user perceptions of Mobile Academy, its influence on interpersonal communication, and ASHA reflections on future mLearning opportunities remains limited. This paper presents the results of a qualitative study exploring these issues in Rajasthan, a state in northern India.

Methods

For peer review only - http://bmjopen.bmj.com/site/about/guidelines.xhtml

ASHA program and training

ASHAs are female CHWs who facilitate antenatal care, institutional deliveries, and immunizations, provide home-based newborn care, promote family planning, hygiene, and nutrition, and convene village health events.[19,20] Current recruitment standards (released in 2012) dictate that ASHAs are required to have completed 10 years of formal education; ASHAs recruited in the first years of the program (2005 – 2012) required only 8 years' education and educational requirements can be "relaxed" in marginalized regions.[21] While some ASHAs have attained education beyond high school, non-literate ASHAs are not uncommon in many states[22]; 17% of ASHAs surveyed in Madhya Pradesh were unable to read a full sentence [23], and 30% of ASHAs surveyed in Uttar Pradesh were low literate.[24] ASHAs have been found to average over 20 work hours a week,[25] and receive a small fixed monthly honorarium as well as performance based remuneration with total monthly income ranging from Rs 900 to Rs 4250 (USD$14 to $65) depending on state-level top up payments and ASHA activity. ASHAs are also compensated Rs 200 (USD$3) for attending face to face trainings. ASHAs have long asserted that they are underpaid.[26,27]

State governments aim to provide ASHAs with 23 days initial training and 10 days supplementary training, as well as monthly supportive supervision through the government primary healthcare system.[28] Early evaluations identified curriculum inadequacies and low quality training delivery as major issues facing the program.[28,29] Over time, trainings have sought to re-orient towards a competency based approach, with efforts to balance health knowledge, clinical skills, and communication and counseling ability.[30] While the program has been credited with bolstering antenatal care, increasing institutional deliveries[31–34] and childhood immunization[35,36] and improving community case management of childhood illness,[37–39] studies have consistently identified gaps in ASHA knowledge and skills including on childhood illness and dangers signs in pregnancy. [37,39–46] Beyond gaps in knowledge and skills, shortcomings in ASHAs' interpersonal and counselling skills have also been identified.[30,47–49] Their communicative and counselling efficacy is influenced by their training on communication and counselling,[48–50] as well as personal characteristics (e.g., the ASHA's confidence, empowerment, education), power hierarchies (e.g., caste, class, religion, gender), other identity and relational factors (e.g., geographic proximity, political affiliations, marital status, number of children, and family relationship histories),[43,51,52] and the health system more broadly (e.g., the extent to which ASHAs are able to linked to clinics that provide high quality health care).[53,54]

Study setting

Rajasthan is a majority Hindi-speaking state in Northern India with a population of 78 million.[55] In 2015, while 85% of men were literate, literacy among women was only 57%.[56] The 2015/16 maternal mortality ratio was 199 per 100,000 live births[57] and the rural under-five mortality rate is 54.4 per 1000 live births.[56] Only 39% of women received the recommended four antenatal care checkups, but 84% of deliveries took place in health facilities.[56] As of January 2018, there were approximately 44,900 ASHAs in the state's rural areas, which is 88% of the targeted recruitment and amounts to 1 ASHA per 1147 rural people.

For peer review only - http://bmjopen.bmj.com/site/about/guidelines.xhtml

Sampling

We received permission to conduct this qualitative study in three districts (Ajmer, Sikar and Pali) of Rajasthan, selected by government authorities. Data collection took place in October and November 2018. ASHAs were selected purposively, using data generated by Mobile Academy's technical system and guidance from block-level ASHA facilitators. We interviewed ASHAs who had completed Mobile Academy when it was officially launched (2016, early 2017), as well as ASHAs who completed Mobile Academy more recently (late 2017, 2018). This decision was made to explore possible differences between early and late completers of Mobile Academy. We also hypothesized that late completers would be more likely to recall specific details of the course, since it would be fresher in their minds. We sought ASHAs with varied Mobile Academy engagement profiles. We considered the number of minutes an ASHA was engaged in the course (while most ASHAs in Rajasthan completed the course in approximately 240 minutes, we sought 11 ASHAs who took 270 minutes or more), their scores (while most ASHAs in Rajasthan had a score in the 40s out of a maximum of 44 points, we sampled 15 ASHAs with scores less than 40/44), and ASHAs who had repeated the course (we sampled four ASHAs who went through the course twice). Our FGDs included a mix of ASHAs by Mobile Academy completion data, nature of Mobile Academy engagement (quiz scores etc.) and education level. We also interviewed district and state level actors (Table 1).

Table 1. Mobile Academy respondent sample

Respondent	Male	Female	Total
In-depth interviews			
• ASHA	0	25	25
• ANM	0	6	6
• ASHA supervisor	3	3	6
• Block ASHA coordinator	2	1	3
• District actor	3	0	3
• State actor	1	1	2
Total	9	36	45
Focus group discussions			
• ASHAs (5 to 6 participants)	0	5	5

Recruitment, data collection and ethics

This study was approved by the institutional ethics review boards at Sigma, Delhi, India (10041/IRB/D/17-18) and JHU, Baltimore, USA (00008360). Interviews and FGDs were conducted by four female qualitative researchers (authors MS, DG, BM and NC), supported by a male research manager (OU) and female research coordinator (KS). All researchers were trained over a one-week period, which included pilot testing the detailed ASHA interview guide, and had master's level social science education or higher. They approached the respondents first by phone to explain the study, identify themselves as working for a Delhi-based research company, explain that they had governmental approval to conduct this study, and ask if they could meet face to face to learn more and, if they agreed, to participate. The interviews took about an hour and were conducted in ASHA homes and in

For peer review only - http://bmjopen.bmj.com/site/about/guidelines.xhtml

other stakeholder's offices and health facilities; the focus group discussions took just over an hour and were conducted in empty school buildings, clinics and courtyards. ASHA family members were often around the home during the ASHA interviews but did not actively listen to or engage with the interviews. Only the researchers and participants were present during the other data collection. The study information and informed consent was read to each potential participant and then summarized in conversational language to ensure comprehension. All respondents provided informed oral consent. Two people who we approached for the study refused: one ASHA who had a family emergency and one state level actor citing lack of interest. All respondents who agreed to participate in the study also allowed audio recording. The research domains explored are presented in box 1.

Box 1. Research domains explored

In-depth interviews

- Relationship with mobile technology (respondents' access to and use of mobile phones);
- Perception of Mobile Academy (voice, pace, content, relevance);
- Mobile Academy uptake (what motivated ASHAs to complete the course, how did they hear about it, did they encounter technological or social barriers to accessing it);
- Mobile Academy quizzes (difficulty, how respondent felt about them, use of the phone keypad to answer questions);
- Mobile Academy completion certificate (process for receiving certificate, importance of certificate);
- Extent to which Mobile Academy is a refresher training versus providing new content;
- Mobile Academy's influence on ASHA-beneficiary interaction and relationships (listening to Mobile Academy with others, telling others about Mobile Academy, drawing from Mobile Academy to help in communication with beneficiaries; the influence of Mobile Academy on ASHAs' confidence, respect and motivation);
- ASHA face-to-face trainings (content, benefits, drawbacks, relationships among ASHAs and between ASHAs and trainers, link to Mobile Academy);
- Desire for further training, especially through mobile technology (content, modality, dose)

Focus group discussions

- General perspectives of Mobile Academy;
- Challenges that ASHAs navigate in trying to promote birth preparedness and family planning;
- Whether Mobile Academy had any influence on how ASHAs spoke to people about these issues;
- ASHA perspectives on future mLearning.

Analysis

Analysis began with daily debrief meetings, wherein the field team (MS, DG, BM, NC, OU and KS) drew from interview notes to discuss emerging themes and adjust elements of the

For peer review only - http://bmjopen.bmj.com/site/about/guidelines.xhtml

guide and sampling based on saturation and emergent sub-topics without the study domains. After data collection, all audio files were transcribed and translated into English and uploaded into Dedoose, a qualitative data management software. Guided by the principles of thematic analysis, KS and OU developed and applied a coding framework with nine code clusters which echoed the topics explored in the interviews and FGDs. The clusters consisted of between three and 11 codes which were primarily drawn from *a priori* areas of interest.

After coding, we generated code reports that enabled us to read all text tagged by the same code. While reviewing these reports we were mindful of the respondent profiles and ASHA-ANM-ASHA facilitator clusters to identify any patterns or trends that existed. We present our findings thematically, grouped under the five components of our framework for understanding CHW engagement with mobile learning courses (Fig 1).

[Figure 1. Framework for understanding CHW engagement with mobile learning courses]

Patient and Public Involvement

The research was shaped by ASHA and other health system actor priorities, experiences and preferences through iterative probing and flexibility within our research domains. Results were disseminated to Government of India stakeholders and actors involved in developing Mobile Academy.

Findings

1. CHW profile

Mobile Academy was considered relevant and appropriate by ASHAs across a wide range of demographic and engagement profiles

While the ASHAs in our five focus groups were selected based only on having completed Mobile Academy and being geographically close to one another, the 25 ASHAs selected for interviews respondent sample was non-typical in several ways (table 2). We sought the rare ASHAs who completed Mobile Academy late (2018 or late 2017), in a state where 93% of ASHAs completed Mobile Academy in 2016 and early 2017. Our late completers (n=12) turned out to be newly recruited ASHAs who were also more educated than early completers (n=13).[21] This selection skewed our respondent sample towards ASHAs with higher education, which is not representative of ASHAs in the state. Whereas only 2 (8%) of the ASHAs interviewed in our study had eight or fewer years of education, over 70% of Rajasthan's ASHAs did.[28] However, our five focus group discussions were composed primarily of "typical" ASHAs (recruited into the ASHA program in the mid-2000s, fewer than 10 years of education, completed Mobile Academy early, and achieved high scores), which enabled us to round out the range of perspectives captured in this research.

For peer review only - http://bmjopen.bmj.com/site/about/guidelines.xhtml

Table 2. ASHA respondents interviewed in this study compared to ASHAs overall in Rajasthan state

Parameter	Our IDI sample n (%) (n=25)	Rajasthan N (%) (n= see notes)
Education		
<=8	2 (8%)	72%*
9 – 10	7 (28%)	23%*
11 – 12	11 (44%)	3%*
Graduate	5 (20%)	1%*
Mobile Academy		
Early completion (2016, early 2017)	13 (52%)	41,164 (93%)**
Late completion (late 2017, 2018)	12 (48%)	1,440 (3%)**
Average quiz score (out of 44 points)	31.6	42.6**
Failed (<22/44)	6 (24%)	91 (0.2%)**
Average duration (minutes)***	291.2	255.0#
ASHAs who repeated the course	3 (12%)	533 (13%)#

*From *ASHA Which Way Forward* (2011) based on a sample of 200 ASHAs
** All ASHAs who completed Mobile Academy in Rajasthan (n=44,545)
*** Of first course. If an ASHA repeated the course, this value would approximately double, but we are not considering this second attempt here, since we report repeats in a separate row.
# Only ASHAs who completed Mobile Academy in Sikar, Pali and Ajmer Districts

We did not identify a qualitative difference between early and late completers in terms of their recall of content or their interpretation of Mobile Academy's appropriateness and ease. Both groups reported that the course was a knowledge assessment and a refresher training, which covered the same content as their core face-to-face trainings. They reported that the course was relevant and appropriate, and easy to understand. ASHAs across the spectrum of education described the quizzes as easy and enjoyable.

We also sought ASHAs for interviews who had lower scores and who went through Mobile Academy more than once. We included six ASHAs who failed (24% of our sample) when only 0.2% of ASHAs in Rajasthan failed Mobile Academy. Surprisingly, the six ASHAs who failed told us that they are unaware that they had performed poorly – despite the fact that the course informs users when they have failed – and, like the ASHAs with high scores, described the course as "easy". All three ASHAs who repeated Mobile Academy (ASHA_11, ASHA_12 and ASHA_24) told us that they did so because their supervisors told them that their first attempt was not properly logged in the system, and that they had to complete the course again. This explanation is likely a miscommunication or misunderstanding because our records show that each attempt was logged.

ASHAs in our sample had universal mobile phone access and variable digital literacy

All the ASHAs in our sample reported having their own phones, which they carried with them when they went out and could use without asking permission. In many households, the ASHA's phone was also used by other family members. Almost half the ASHAs in our sample reported that they used a smart phone, which indicates a more digitally-enabled group of ASHAs compared to the norm; in Madhya Pradesh a recent survey found that only 11% of ASHAs had a smartphone.[58] Even among our savvy qualitative sample, internet access was variable since few ASHAs bought mobile internet data regularly. (Internet access

had no bearing on Mobile Academy access because Mobile Academy is a dial-in mobile phone service.) ASHAs explained that the government-issued SIM for ASHA-related activities allowed them to speak to other ASHAs and ASHA facilitators and make calls to beneficiaries; however in some areas this government SIM did not work because of network issues or late payment of monthly bills by the government. ASHAs kept a second, personal, SIM, which they sometimes used for ASHA work as well.

Some ASHAs reported receiving calls from government actors, including their ANM and ASHA facilitator, as well as health system actors in district headquarters, the state capital and Delhi. These calls were to inform them about trainings and meetings, ask ASHAs to report data such as the number of pregnancies, or, in one case, to ask the ASHA to follow up with specific high risk patients. Others had not received any ASHA-work related phone calls. Over half of the ASHAs in this qualitative sample were on WhatsApp, compared to 12% in a representative sample of ASHAs in Madhya Pradesh.[58] Our ASHAs reported using WhatsApp to share reports and information with and among ASHAs, however access to WhatsApp was irregular and many ASHAs explained that their husbands were actually in the ASHA WhatsApp groups but reported information from the WhatsApp groups back to them.

All ASHAs were confident that they could receive calls from beneficiaries about emergencies, such as women in labor, and could make outgoing calls to the ambulance and to families, often to notify them about vaccinations and health checkups. Beyond receiving and making calls, most ASHAs self reported that they could open and read SMS and WhatsApp messages but several disclosed that they got stuck sometimes doing these activities. Our research team did not test these capacities. Most said that they were not able to write and send messages by SMS or in WhatsApp, even among those who completed education beyond 12th standard: "Neither do I know how to send SMS nor do I send. It is not required." (ASHA_08, 44/44, graduate). Several ASHAs described needing help from family members to operate their phones, including saving contacts and opening and reading messages.

2. Program access and uptake

Mobile Academy was widely understood to be primarily an examination

ASHAs and higher-level health system stakeholders described Mobile Academy as an ASHA knowledge test or examination rather than a refresher course. Relatedly, several higher level stakeholders (DIST_01, BLCK_01, BLCK_02) considered Mobile Academy to be an assessment of the ASHAs and were concerned that Mobile Academy could be completed by other people or that ASHAs could cheat by sharing the answers to the quizzes.

ASHAs completed Mobile Academy because of pressure from above, but also expressed desire to gain knowledge

ASHAs described pressure to complete Mobile Academy "*aage se* [from above]." They repeatedly reported being told by their supervisors that completing the course was *zaroori* [necessary/essential] and were also aware that the ANM and ASHA supervisor were under pressure from district level authorities to have all their ASHAs finish Mobile Academy.

For peer review only - http://bmjopen.bmj.com/site/about/guidelines.xhtml

> There was pressure, it's important to do it… we were getting messages, phone calls, again and again, that this is important for you to do. (FGD_ASHA_03)

A few ASHAs described being worried that they would lose their positions if they did not complete Mobile Academy or if they did not pass. One ASHA also reported being told that ASHA pay would increase upon completing Mobile Academy, which was not the case (FGD_ASHA_03). ASHAs were not paid for completing Mobile Academy, and when asked about payment they tended to say it was not necessary. Instead, they framed the course was a means to remain in their positions and excel at their work. It was only when discussing future mLearning opportunities (section 5) that several ASHAs mentioned that they ought to be paid for these activities.

In addition to being required to take the course, ASHAs described being motivated to completed Mobile Academy by their desire to gain more knowledge, as well as general curiosity, an interest in receiving the certificate and a desire to show higher level health system actors that they are knowledgeable.

> We get to learn, that it the reason. There is a will to learn, as an ASHA - for ourselves and for the villagers, like our family, like our village (ASHA_15, 43/44, 12th)

Mobile Academy uptake among our sample did not follow planned dose and frequency

While ASHAs could call into Mobile Academy and proceed through the course whenever they had time over weeks or even months, the ASHAs we spoke to reported completing it rapidly in just one or two days. National level data on Mobile Academy engagement found that 36% of ASHAs completed the course within three days, and 12% within the first 24 hours.[59] Our data can offer insight into what might have driven this sizable minority of ASHAs to proceed so rapidly. Some of the ASHAs in our sample reported not knowing that the course could be completed in parts and some were unsure how much time could elapse between starting and completing the course. Others worried about not being able to get through to the Mobile Academy line if they called back later. Many also emphasised that they were under pressure to complete the course quickly.

3. Experience of Mobile Academy

Initial fear replaced by enjoyment; ASHAs praised the pace and tone, valued repetition

When ASHAs first heard that they were to complete a training by mobile phone, they described feeling nervous. They did not know what it would entail or were concerned about having to provide answers to quiz questions. However, they quickly felt comfortable with both the technology and content. ASHAs reported enjoying the course because of the warm

For peer review only - http://bmjopen.bmj.com/site/about/guidelines.xhtml

and friendly tone, option to repeat content, and the fact that the quiz questions covered familiar topics and were easy.

> At first, I was very scared that I would give the wrong answer. Then when I finished it, I felt very good. (FGD_ASHA_05)

> I thought I do not know what she will ask and what she will not ask. This was the fear. But after listening I was not afraid. (ASHA_23, 38/44, 10th)

ASHAs expressed very positive overall opinions of the course. The character voices were frequently described as loving, warm and calm: "Sweetness was there in the voice" (ASHA_24, 44/44, 12th, early adopter). The Hindi language speech was easy to understand for the ASHAs, although they generally spoke Marwadi, a Rajasthani dialect close to Hindi.

> Her voice was good and sweet. […] She spoke exactly spoke like us. (ASHA_29, 35/44, 12th)

> We could trust her well. She was talking in such a way that it was nobody else but someone of her own, apna hi hai [like she knows us]. (ASHA_15, 43/44. 12th)

> They explained with a lot of love, very slowly. And asked [questions] calmly. (FGD_ASHA_03)

ASHAs felt the pace was appropriate: "neither it was fast nor slow; everything was explained in a proper manner" (ASHA_29) and greatly valued being able to repeat content. ASHAs emphasized that the repetitive content and ability to re-listen to lessons was a major strength of the program: "they were repeating some of the topics two to three times and then we were able to remember that nicely" (ASHA_31, 21/44, 8th).

Quizzes increased ASHA confidence

As designed, the ASHAs experienced quizzes to be easy but enjoyable. Although described as "easy" by all, ASHAs still said they had to pay attention properly in order to complete them.

> ASHA1: [I] got 100 out of 100. I felt like, if more like these are happening, we will do it.
> ASHA2: We had a lot of fun, lot of fun
> ASHA3: That we cleared the paper [passed the test]! If we get the next paper [test], we will clear that too.
> ASHA2: We passed it easily. We didn't have to listen to anyone scolding us.
> [all laugh]
> ASHA4: I felt like I passed a competition
> ASHA3: I was so happy to get good marks (FGD_ASHA_03)

> Absolutely we have to keep our mind on one point. Whatever they tell we have to find out the answer from that immediately. We must not delay. (ASHA_24, 44/44, 12th)

As mentioned above, those with low quiz scores were unaware that there was any issue with their score and described the quizzes as "easy". None reported receiving feedback that they had failed, despite the fact that the course reports back to users whenever they select

For peer review only - http://bmjopen.bmj.com/site/about/guidelines.xhtml

an incorrect quiz answer and if they have failed at the end, suggesting that ASHAs forgot, did not realize, or did not want to admit to our research team that they had struggled with the quiz.

Mobile Academy is often a social activity

Despite the individual dial-in design, many ASHAs reported involving other people. Some (ASHA_31, FGD_ASHA_05) reported proceeding through Mobile Academy with a group of other ASHAs during group training sessions. Some played the content for colleagues, such as anganwadi workers and ASHA supervisors, to show them what the course was about or to seek assistance. Others played Mobile Academy on speakerphone for their children or in-laws to explain why they were on the phone for a long duration and to share the content.

Mobile Academy a complement to face-to-face training

ASHAs greatly valued that mobile phone-based training allowed them to complete the course from home, when they had spare time. This home-based model avoided the difficulties of making childcare arrangements and leaving behind domestic responsibilities inherent to face-to-face training or meetings.

> Otherwise [for face-to-face training] we have to leave the family and children and go. We have tension in that case. But here we can sit back at home and do the training. We can do it whenever we get time. (ASHA_03, 44/44, 10th)

Furthermore, ASHAs in remote areas valued that mLearning did not require the time and discomfort of travelling long distances to reach training centres. Two ASHAs (ASHA_11, ASHA_18) also noted that they could focus better alone at home compared to group settings where multiple people were speaking at once.

> We can listen to it peacefully. There, [face-to-face training] ten people are sitting. Who is saying what? Here we can listen comfortably. (ASHA_11, 42/44, 8th)

Several ASHAs contrasted the kind and encouraging approach of Mobile Academy with the punitive and demoralizing approach to training and supervision that they experienced face-to-face. ASHAs reported being frightened of supervisors and trainers, explaining that no ASHAs speak because “we feel a bit scared”, “if we are wrong he will scold us” (FGD_ASHA_03); “they scold us badly” (FGD_ASHA_05). However, other ASHAs found face-to-face trainers to be supportive and noted that in-person training offered the opportunity to ask questions whenever they did not understand; to learn techniques and practices by seeing them demonstrated; to connect with other ASHAs (“all the ASHAs get together, they talk and gossip” (FGD_ASHA_05)), trainers and facilitators; and to get out of the house.

> ASHA1: Sister, it’s like, in training face to face we will get to learn more. […] In Mobile Academy, we can only listen. In the other one, we will get to learn –
> ASHA2: The way/technique
> ASHA1: Practical
> ASHA3: They show us
> ASHA2: In face to face training, madam, if we ask questions we also get answers. We can ask also, what, why is this. In that [Mobile Academy], we can't even ask. We can just say yes or no. (FGD_ASHA_04)

For peer review only - http://bmjopen.bmj.com/site/about/guidelines.xhtml

Most ASHAs explained that both modalities of training had benefits and drawbacks, and thus hoped to receive further face-to-face training, supplemented with mLearning.

4. Mobile Academy's influence on ASHAs

Mobile Academy showcased a positive communication style that ASHAs can apply when speaking to beneficiaries

When asked about Mobile Academy's influence, ASHA most commonly explained that the course changed how they communicated with beneficiaries. They explained that they modelled the tone used by the voice actors in Mobile Academy to speak to beneficiaries, introducing a more loving and calm approach to their communication with women:

> [We] learned how to speak with *prem* [love] (laughs) […] After doing Mobile Academy, we've made some changes in the way we talk. […] This only: explain once. If they don't understand then explain again, but explain with love. Don't get angry, that won't work there. (FGD_ASHA_03)

They spoke of internalizing the importance of taking time to talk to beneficiaries, not rushing, and repeating content in a caring manner.

Mobile Academy increased ASHA confidence and reinforced knowledge

ASHAs reported feeling more confident and knowledgeable after completing Mobile Academy. As noted above, Mobile Academy was designed as a refresher training that covered the same topics as face-to-face training; ASHAs reported experiencing it in this way and said that the refresher content enabled them to retain some specific knowledge.

> If they tell us again, it makes a difference. […] We are able to understand [now] that the training happened for Mobile Academy. The course refreshes things. [Face-to-face] trainings happened here 8 to 10 years ago." (FGD_ASHA_03)

While ASHAs reported that they did not tell beneficiaries that they completed Mobile Academy and did not listen to Mobile Academy with beneficiaries, they drew from their training, including Mobile Academy, to answer beneficiary questions.

> Earlier we did not have courage to talk in front of others. Now we have courage. If we did not know anything, what would we tell others? If we have a little bit of knowledge only then we can tell others. If anybody asks anything, we can answer them as well. (ASHA_15, 43/44, 12th)

While ASHAs were to receive Mobile Academy completion certificates, many did not. ASHAs who had not received a certificate did not articulate personal disappointment or concern about community perception. However, some worried that they lacked proof to show health system stakeholders that they completed the course.

For peer review only

5. Future training

ASHAs and higher-level health system actors were very positive about integrating future mLearning alongside face-to-face training to reinforce, refresh, or deepen understanding. ASHAs wanted future Mobile Academy content to cover newborn care, family planning, nutrition, non-communicable disease (diabetes, hypertension and cancer), adolescent health, village health committees, tuberculosis, malaria, leprosy, and cataracts. They said that future mobile training could consist of up to six hours of content, spread in half hour increments over several weeks or months. When asked directly about incentives to complete future trainings most but not all ASHAs requested payment. One noted that they are paid for face-to-face trainings and said that mLearning ought not to be different: “We get money for every training. So if we get in this one, it is good.” (ASHA_11) However, several said that no money was needed, explaining that the knowledge itself is desirable (“Because we have to learn something, so why should they pay money?” ASHA_29) and instead requested other incentives such as certificates or the opportunity for promotion.

Discussion

This qualitative exploration of Mobile Academy found strong positive perceptions of the course among ASHAs and other health system actors. ASHAs were driven to take the course because of a desire to learn as well as pressure from supervisors. They felt that the warm and friendly tone and their ability to repeat content refreshed their knowledge and showcased a positive communication style that they could apply to interactions with beneficiaries. Respondents were enthusiastic about future mLearning, although they noted that face-to-face training could not be replaced with mobile phone-based programs. Furthermore, respondents noted that if mLearning were to become a repeated and regular part of ASHA training, financial incentives would be required to compensate ASHAs for their time and sustain motivation.

In addition to these outcomes, several surprising aspects of ASHA engagement with the course emerged. First, although Mobile Academy was designed for ASHAs with eight years of education, ASHAs with 12 or more years of education also said that they valued the course. As ASHA educational requirements and mobile phone savvy increase, research examining whether some ASHAs can handle more complex material, alternative teaching styles, and the more sophisticated digital engagement used elsewhere will be valuable.[60–62] Nonetheless, given the persistence of low literacy and limited digital skills among women in India[63] including ASHAs[23,24,64], ongoing investment in audio based trainings that can be accessed from any phone, rather than textual or smartphone based programming, remains critical.

Second, although Mobile Academy was designed to be a training program with simple quiz questions that served only to motivate and engage ASHAs, the quiz component became the focus. Further research is needed to understand the range of effective ways to engage interactive feedback as motivators, teaching tools, and systems for assessment and accreditation. Third, the ASHAs in our sample tended to complete the course rapidly, in one or two long sessions, rather than spreading the content over many weeks. Nationally, a

For peer review only - http://bmjopen.bmj.com/site/about/guidelines.xhtml

smaller portion (36%) took a rapid approach.[59] The ASHAs in our qualitative study explained that they would have liked to proceed through the course more slowly, but took the rapid approach because of pressure from supervisors and concern that they might be unable to access the course again later. This finding, as well as the outsized focus on the quizzes, suggests that supervisors and FLHWs require steady communication about the intent and engagement options for mLearning programs.

Fourth, ASHAs and health system actors understood Mobile Academy as an internal accreditation rather than an achievement that could bolster community trust. Other studies from South Asia have found that women's engagement with mobile phones enables "modern" aspirations for education and socially mobility but requires reputational risk management.[63,65] The ways in which ASHA use of digital technology affects their social identities and relationships warrants further research.

Power hierarchies in the Indian health system (e.g., by education, caste, professional status, gender, urban-rural residence) can be reproduced in trainer-ASHA dynamics and in ASHA-beneficiary interactions, ultimately limiting communication and trust throughout the system.[54,66] ASHAs reported that Mobile Academy's lasting impact on them was both informational and affective – ASHAs spontaneously and frequently focused on the "loving" communication style and their intention to incorporate this into their interactions with beneficiaries. A few ASHAs contrasted the kind tone in Mobile Academy to a more intimidating teaching style used at some face-to-face trainings. And although many ASHAs expressed sympathy towards families that did not abide by their recommendations, others expressed frustration. ASHAs noted persistent barriers to change, including resistance from mothers-in-law, fear of poor quality care and financial costs at hospitals and shame and secrecy around reproduction. But several ASHAs described families that did not adhere to their guidance as stubborn, lazy and careless, and felt little shared identity the populations they served. The capacity for digital trainings to improve communication within this hierarchical space require further exploration: To what extent does Mobile Academy subvert hierarchies versus engage with them in a productive manner? Can face-to-face trainers learn from Mobile Academy's tone and approach? And what is the actual impact of this "loving" tone on ASHA-beneficiary interactions? Ultimately, it is important to note that strengthening ASHA-beneficiary relationships will require ongoing ASHA capacity building (of which mLearning can play a role), community engagement, and quality improvements in the government health services themselves.[67]

This study was limited to the experiences of ASHAs in the Indian state of Rajasthan. Given that Mobile Academy has been scaled up across 13 states, additional localized research would be beneficial. Another limitation of this study is that we only present ASHA and supervisor perspectives on how Mobile Academy influenced ASHA knowledge and communication with beneficiaries. Evaluating these assertions was beyond the scope of our research but would be very beneficial. Finally, the time lag between when ASHAs took Mobile Academy (between 2016 and early 2018) and when we interviewed them about it (late 2018) may have reduced the emotional intensity of their responses or introduced inaccurate recollections of program components.

For peer review only - http://bmjopen.bmj.com/site/about/guidelines.xhtml

Conclusion

While mLearning programs have been found capable of improving aspects of FLHW knowledge, patient engagement, and supervision, they tend to require smartphone technology and user digital skills that remain out of reach to many of India's rural FLHWs.[38,39] Many also rely on trainer inputs such as remote moderation and feedback[10], that are challenging add-ons in overstretched systems such as India's where vacancies throughout the supervisory hierarchy remain an issue.[68] Mobile Academy required only a basic phone and the ability to use a keypad to access its pre-recorded content and interactive quiz questions. It was well received by the ASHA FLHWs in our sample, which included a wide range of education levels and experience, including some ASHAs with more education and better digital access than the norm. Policymakers can note Mobile Academy's high acceptability among users, as well as its potential to not only reinforce knowledge but also encourage positive beneficiary-FLHW communication style. These findings position the Mobile Academy model as a viable mLearning option for many LMIC contexts to reinforce ongoing face-to-face training.

Acknowledgements

The authors are grateful to the ASHAs and other health system actors who generously provided their time and insights to make this research possible. This work was made possible by the Bill and Melinda Gates Foundation. We thank Diva Dhar, Suhel Bidani, Rahul Mullick, Dr. Suneeta Krishnan, Dr. Neeta Goel and Dr. Priya Nanda for believing in us and giving us this opportunity. We additionally thank Vinit Pattnaik at OPM and Erica Crawford at Johns Hopkins for their support to the financial management of our work.

Data sharing

Data for this study consist of qualitative interview and focus group discussion transcripts. Uploading all transcripts for open availability would compromise our ability to fully mask participant details. However, we are happy to share anonymized portions of these transcripts upon reasonable request.

Competing interests

All authors have completed the Unified Competing Interest form (available on request from the corresponding author) and declare that the research reported was funded by the Bill and Melinda Gates Foundation. SC is employed by BBC Media Action, one of the entities supporting program implementation for Mobile Academy. The authors do not have other relationships and are not engaged in activities that could appear to have influenced the submitted work.

For peer review only

Contributor statement

KS, SC , OU and AEL conceptualized and designed the study. MS, DG, BM and NC conducted the data collection and preliminary data analysis through daily analytic debriefs. KS and OU led and managed the data collection and analysis, including the coding and thematic synthesis. KS drafted the manuscript and revised it based on critical and substantive input from SC, OU, AEL, MS, BM and NC. All authors agree to be accountable for all aspects of the work related to accuracy and integrity.

Funding statement

This work was supported by the Bill and Melinda Gates Foundation grant number OPP1179252.

For peer review only

Works Cited

1. WHO. The World Health Report 2008. Primary Health Care - Now more than ever [Internet]. Vol. 26, The World Health Report. Geneva, Switzerland; 2008. Available from: http://www.who.int/whr/2008/whr08_en.pdf
2. WHO. Global Strategy on Human Resources for Health: Workforce 2030 [Internet]. Synthesis Paper of the Thematic Working Groups. Geneva; 2016. Available from: http://www.who.int/hrh/resources/pub_globstrathrh-2030/en/
3. Scott K, Beckham S, Gross M, Pariyo G, Rao K, Cometto G, et al. What do we know about community-based health programs? A systematic review of existing reviews on community health workers and their integration with health systems. Hum Resour Health. 2018;1–17.
4. Cometto G, Ford N, Pfaffman-Zambruni J, Akl EA, Lehmann U, McPake B, et al. Health policy and system support to optimise community health worker programmes: an abridged WHO guideline. Lancet Glob Heal [Internet]. 2018;6(12):e1397–404. Available from: http://dx.doi.org/10.1016/S2214-109X(18)30482-0
5. Marcolino MS, Oliveira JAQ, D'Agostino M, Ribeiro AL, Alkmim MBM, Novillo-Ortiz D. The Impact of mHealth Interventions: Systematic Review of Systematic Reviews. JMIR mHealth uHealth [Internet]. 2018 Jan 17;6(1):e23–e23. Available from: https://www.ncbi.nlm.nih.gov/pubmed/29343463
6. Agarwal S, Perry HB, Long LA, Labrique AB. Evidence on feasibility and effective use of mHealth strategies by frontline health workers in developing countries: Systematic review. Trop Med Int Heal. 2015;20(8):1003–14.
7. O'Donovan J, Bersin A, O'Donovan C. The effectiveness of mobile health (mHealth) technologies to train healthcare professionals in developing countries: a review of the literature. BMJ Innov [Internet]. 2015 Jan;1(1):33–6. Available from: http://innovations.bmj.com/lookup/doi/10.1136/bmjinnov-2014-000013
8. O'Donovan J, O'Donovan C, Kuhn I, Sachs SE, Winters N. Ongoing training of community health workers in low-income and middle-income countries: a systematic scoping review of the literature. BMJ Open [Internet]. 2018 Apr 1;8(4):e021467. Available from: http://bmjopen.bmj.com/content/8/4/e021467.abstract
9. Schoeman F. Digital Tools for Training Frontline Health Workers in Low- and Middle-Income Countries: A Systematic Review. Univeristy of Cape Town; 2019.

10. Henry JV, Winters N, Lakati A, Oliver M, Geniets A, Mbae SM, et al. Enhancing the Supervision of Community Health Workers With WhatsApp Mobile Messaging: Qualitative Findings From 2 Low-Resource Settings in Kenya. Glob Heal Sci Pract [Internet]. 2016 Jun 20;4(2):311–25. Available from: http://www.ghspjournal.org/lookup/doi/10.9745/GHSP-D-15-00386
11. Joos O, Silva R, Amouzou A, Moulton LH, Perin J, Bryce J, et al. Evaluation of a mHealth Data Quality Intervention to Improve Documentation of Pregnancy Outcomes by Health Surveillance Assistants in Malawi: A Cluster Randomized Trial. Krukowski R, editor. PLoS One [Internet]. 2016 Jan 5;11(1):e0145238. Available from: https://dx.plos.org/10.1371/journal.pone.0145238
12. Vallières F, McAuliffe E, Van Bavel B, J. Wall P, Trye A. There's No App for That: Assessing the Impact of mHealth on the Supervision, Motivation, Engagement, and Satisfaction of Community Health Workers in Sierra Leone. Ann Glob Heal [Internet]. 2017 Mar 8;82(5):936. Available from: https://annalsofglobalhealth.org/article/10.1016/j.aogh.2016.07.002/
13. Ward VC, Raheel H, Weng Y, Mehta KM, Dutt P, Mitra R, et al. Impact Of Mhealth Interventions for Reproductive, Maternal, Newborn and Child Health and Nutrition at Scale: Bbc Media Action and The Ananya Program in Bihar, India. J Glob Health. 2020;10(2):1–13.
14. Carmichael SL, Mehta K, Srikantiah S, Mahapatra T, Chaudhuri I, Balakrishnan R, et al. Use of mobile technology by frontline health workers to promote reproductive, maternal, newborn and child health and nutrition: A cluster randomized controlled trial in Bihar, India. J Glob Health. 2019;9(2).
15. Diedhiou A, Gilroy KE, Cox CM, Duncan L, Koumtingue D, Pacque-Margolis S, et al. Successful mLearning pilot in Senegal: Delivering family planning refresher training using interactive voice response and SMS. Glob Heal Sci Pract. 2015;3(2):305–21.
16. Bellina L, Azzolina G, Nucatola I, Maggio A, Consiglio F, Ingrassia R. m-phone impact on practical training: role of m-phone as part of the educative method for training local workers of rural areas of developing countries. Health Technol. 2014;4:289–94.
17. Limaye R, Deka S, Ahmed N, Mwaikambo L. Designing eLearning courses to meet the digital literacy needs of healthcare workers in lower- and middle-income 71 countries: Experiences from the Knowledge for Health Project. Knowl Manag E-Learning. 2015;7(4):601–15.
18. Pimmer C, Mbvubdula F. One message, many voices: Mobile audio counselling in health education. J Health Care Poor Underserved. 2018;29(1):463–80.
19. MoHFW India. National Rural Health Mission (2005-2012): Mission Document [Internet]. New Delhi; 2005. Available from: http://mohfw.nic.in/NRHM/Documents/NRHM Mission Document.pdf
20. MoHFW India. ASHA Guidelines. New Delhi: National Rural Health Mission, Ministry of Health and Family Welfare, Government of India; 2006.
21. Jhalani M. Education qualification 10th standard for ASHA selection 22.08.2012. New Delhi: Ministry of Health and Family Welfare, Government of India; 2012.
22. MoHFW. Ninth Common Review Mission Report 2015. Ministry of Health and Family Welfare, Government of India; 2015.
23. Mohan D, Shah N, Scott K, Ummer O, Chamberlain S, Lefevre AE. ASHA competency assessment in Madhya Pradesh. forthcoming.
24. DeRenzi B, Wacksman J, Dell N, Lee S, Lesh N, Borriello G, et al. Closing the Feedback

For peer review only - http://bmjopen.bmj.com/site/about/guidelines.xhtml

Loop: A 12-month Evaluation of ASTA, a Self-Tracking Application for ASHAs. Proc Eighth Int Conf Inf Commun Technol Dev [Internet]. 2016;22:1--22:10. Available from: http://doi.acm.org/10.1145/2909609.2909652
25. MoHFW. Update on the ASHA Programme, January 2017 [Internet]. New Delhi, India; Available from: http://nhsrcindia.org/sites/default/files/Update on ASHA Programme- January-2017.pdf
26. Bhatia K. Community health worker programs in India: a rights-based review. Perspect Public Health. 2014;134(5):276–82.
27. Bhatia K. Performance based incentives of the ASHA Scheme: Stakeholders' Perspectives. Econ Polit Wkly. 2014;49(22):145.
28. NHSRC. ASHA: Which way forward? Evaluation of the ASHA Programme. Mission NRH, editor. New Delhi: National Health Systems Resource Centre; 2011.
29. Nirupam B, Dholakia RH. Improving the performance of Accredited Social Health Activists in India. Working Paper No. 1. Mumbai: Columbia Global Centers | South Asia, Columbia University; 2011.
30. Shrivastava A, Srivastava A. Measuring communication competence and effectiveness of ASHAs (accredited social health activist) in their leadership role at rural settings of Uttar Pradesh (India). Leadersh Heal Serv [Internet]. 2016;29(1):69–81. Available from: http://www.emeraldinsight.com/doi/10.1108/LHS-12-2014-0079
31. Gopalan SS, Durairaj V. Addressing maternal healthcare through demand side financial incentives: experience of Janani Suraksha Yojana program in India. BMC Health Serv Res [Internet]. 2012;12(1):319. Available from: http://www.scopus.com/inward/record.url?eid=2-s2.0-84867562946&partnerID=tZOtx3y1
32. Malini S, Tripathi RM, Khattar P, Nair KS, Tekhre YL, Dhar N, et al. A Rapid Appraisal on Fuctioning of janani Suraksha Yojana in South Orrisa. Heal Poluplation. 2008;31(2):126–31.
33. Sidney K, Tolhurst R, Jehan K, Diwan V, De Costa A. "The money is important but all women anyway go to hospital for childbirth nowadays" - a qualitative exploration of why women participate in a conditional cash transfer program to promote institutional deliveries in Madhya Pradesh, India. BMC Pregnancy Childbirth [Internet]. 2016;16(1):47. Available from: http://www.pubmedcentral.nih.gov/articlerender.fcgi?artid=4779242&tool=pmcentrez&rendertype=abstract
34. Padda P, Devgun S, Gupta V, Chaudhari S, Singh G. Role of ASHA in improvement of maternal health status in northern India: An urban rural comparison. Indian J Community Heal. 2013;25(4):465–71.
35. Mony P, Raju M. Evaluation of ASHA programme in Karnataka under the National Rural Health Mission. BMC Proc [Internet]. 2012;6(Suppl 5):P12. Available from: http://www.biomedcentral.com/1753-6561/6/S5/P12
36. Wagner AL, Bettampadi D, Porth JM, Boulton ML. The impact of increased use of ASHAs on rural immunization coverage in India. 17th Int Congr Infect Dis [Poster] [Internet]. 2016;45:209–10. Available from: http://dx.doi.org/10.1016/j.ijid.2016.02.478
37. Awasthi S, Nichter M, Verma T, Srivastava NM, Agarwal M, Singh JV, et al. Revisiting community case management of childhood pneumonia: Perceptions of caregivers and grass root health providers in Uttar Pradesh and Bihar, Northern India. PLoS One.

For peer review only - http://bmjopen.bmj.com/site/about/guidelines.xhtml

2015;10(4):1–19.

38. Bhandari N, Mazumder S, Taneja S, Sommerfelt H, Strand TA, Group ES. Effect of implementation of Integrated Management of Neonatal and Childhood Illness (IMNCI) programme on neonatal and infant mortality: cluster randomised controlled trial. BMJ. 2012;1634(March):1–13.

39. Mohan P, Kishore B, Singh S, Bahl R, Puri A, Kumar R. Assessment of implementation of integrated management of neonatal and childhood illness in India. J Heal Popul Nutr [Internet]. 2011;29(6):629–38. Available from: http://www.ncbi.nlm.nih.gov/pubmed/22283037

40. Mishra A. The role of the Accredited Social Health Activists in effective health care delivery: evidence from a study in South Orissa. BMC Proc [Internet]. 2012;6(Suppl 1):1–2. Available from: http://search.ebscohost.com/login.aspx?direct=true&db=aph&AN=71341372&site=ehost-live

41. Shrivastava SR, Shrivastava PS. Evaluation of trained Accredited Social Health Activist (ASHA) workers regarding their knowledge, attitude and practices about child health. Rural Remote Health. 2012;12(4):1–7.

42. Sinha LN, Kaur P, Gupta R, Dalpath S, Goyal V, Murhekar M. Newborn care practices and home-based postnatal newborn care programme - Mewat, Haryana, India, 2013. West Pacific Surveill response J WPSAR [Internet]. 2014;5(3):Russmann, W., Konig, U., Schlimbach, K., Pawlowska. Available from: http://www.scopus.com/inward/record.url?eid=2-s2.0-84965191350&partnerID=tZOtx3y1

43. Sharma R, Webster P, Bhattacharyya S. Factors affecting the performance of community health workers in India: A multi-stakeholder perspective. Glob Health Action. 2014;7(1):1–8.

44. Ramadurg U, Vidler M, Charanthimath U, Katageri G, Bellad M, Mallapur A, et al. Community health worker knowledge and management of pre-eclampsia in rural Karnataka State, India. Reprod Health [Internet]. 2016;13(Suppl 2):113. Available from: http://www.ncbi.nlm.nih.gov/pubmed/27719678

45. Bansal SC, Nimbalkar SM, Shah NA, Shrivastav RS, Phatak AG. Evaluation of knowledge and skills of home based newborn care among Accredited Social Health Activists (ASHA). Indian Pediatr. 2016;53(8):689–91.

46. Kori S, Bhatia M, Mishra A. A cross-sectional assessment of knowledge of ASHA workers. J Krishna Inst Med Sci Univ [Internet]. 2015;4(4):57–63. Available from: http://www.jkimsu.com/jkimsu-vol4no4/JKIMSU, Vol. 4, No. 4, Oct-Dec 2015 Page 57-63.pdf%5Cnhttp://ovidsp.ovid.com/ovidweb.cgi?T=JS&PAGE=reference&D=emed13&NEWS=N&AN=2015482227

47. Das E, Panwar DS, Fischer EA, Bora G, Carlough MC. Performance of accredited social health activists to provide home-based newborn care: A situational analysis. Indian Pediatr. 2014;51(2):142–4.

48. Panwar DS, Naidu V, Das E, Verma S, Khan AA. Strengthening support mechanisms for Accredited Social Health Activists in order to improve home-based newborn care in Uttar Pradesh, India. BMC Proc [Internet]. 2012;6(Suppl 5):1–2. Available from: http://search.ebscohost.com/login.aspx?direct=true&db=aph&AN=82432034&site=ehost-live

For peer review only - http://bmjopen.bmj.com/site/about/guidelines.xhtml

49. Goel AD, Gosain M, Amarchand R, Sharma H, Rai S, Kapoor SK, et al. Effectiveness of a Quality Improvement Program Using Difference-in-Difference Analysis for Home Based Newborn Care – Results of a Community Intervention Trial. Indian J Pediatr [Internet]. 2019;86(11):1028–35. Available from: http://www.embase.com/search/results?subaction=viewrecord&from=export&id=L2002309415%0Ahttp://dx.doi.org/10.1007/s12098-019-03012-4
50. Smittenaar P, Ramesh BM, Jain M, Blanchard J, Kemp H, Engl E, et al. Bringing Greater Precision to Interactions Between Community Health Workers and Households to Improve Maternal and Newborn Health Outcomes in India. Glob Heal Sci Pract [Internet]. 2020 Sep 30;8(3):358–71. Available from: http://www.ghspjournal.org/lookup/doi/10.9745/GHSP-D-20-00027
51. Scott K, George AS, Harvey SA, Mondal S, Patel G, Ved R, et al. Beyond form and functioning: Understanding how contextual factors influence village health committees in northern India. PLoS One. 2017 Aug;12(8):e0182982.
52. Sarin E, Lunsford SS. How female community health workers navigate work challenges and why there are still gaps in their performance: A look at female community health workers in maternal and child health in two Indian districts through a reciprocal determinism framework. Hum Resour Health. 2017;15(1).
53. Ved R, Scott K. Counseling is a relationship not just a skill: Re-conceptualizing health behavior change communication by india's accredited social health activists. Glob Heal Sci Pract. 2020;8(3):332–4.
54. Scott K, Shanker S. Tying their hands? Institutional obstacles to the success of the ASHA community health worker programme in rural north India. AIDS Care. 2010;22 Suppl 2(931159996):1606–12.
55. UIA India. State/UT wise Aadhaar Saturation. 2018.
56. MoHFW India. National Family Health Survey (NFHS-4), India, 2015-16: Rajasthan [Internet]. Mumbai; 2017. Available from: http://www.rchiips.org/nfhs
57. NITI Aayog. Maternal Mortality Ratio (MMR) (per 100000 live births), Rajasthan, 2014-2016 [Internet]. Maternal Mortality Ratio: India, EAG& Assam, Southern States and Other States (per 100000 live births). 2019 [cited 2019 Dec 20]. Available from: https://niti.gov.in/content/maternal-mortality-ratio-mmr-100000-live-births
58. Lefevre AE, Mohan D, Scott K, Chamberlain S. Swipe out: The digital skill gap between men and women in rural Madhya Pradesh. to be Submitt.
59. Bashingwa JJH, Shah N, Mohan D, Scott K, Chamberlain S, Mulder N, et al. Examining Reach and Exposure of a Mobile Phone-Based Training Program for frontline health workers (ASHAs) in India. BMJ Glob Heal.
60. Amoakoh-Coleman M, Borgstein ABJ, Sondaal SFV, Grobbee DE, Miltenburg AS, Verwijs M, et al. Effectiveness of mHealth interventions targeting health care workers to improve pregnancy outcomes in low- and middle-income countries: A systematic review. J Med Internet Res. 2016;18(8):1–18.
61. P. L, R. R, G.C.Y. L, G. D, Ž. C, J. C. Influences on the Implementation of Mobile Learning for Medical and Nursing Education: Qualitative Systematic Review by the Digital Health Education Collaboration. J Med Internet Res [Internet]. 2019;21(2):e12895. Available from: http://www.embase.com/search/results?subaction=viewrecord&from=export&id=L626580093%0Ahttp://dx.doi.org/10.2196/12895
62. Tudor Car L, Riboli-Sasco EF, Marcano Belisario JS, Nikolaou CK, Majeed A, Zary N, et

For peer review only

al. Mobile learning for delivering health professional education. Cochrane Database Syst Rev. 2018;2018(6).
63. Scott K, Shinde A, Ummer O, Yadav S, Sharma M, Purty N, et al. Freedom within a cage: How patriarchal gender norms limit women's use of mobile phones in rural central India. BMJ Glob Heal. 2021;6.
64. MoHFW. Fifth Common Review Mission Report 2011. National Rural Health Mission, Ministry of Health and Family Welfare, Government of India; 2011.
65. Huang JQ. Digital aspirations: 'wrong-number' mobile-phone relationships and experimental ethics among women entrepreneurs in rural Bangladesh. J R Anthropol Inst. 2018;24(1):107–25.
66. Roalkvam S. Health governance in India: citizenship as situated practice. Glob Public Health [Internet]. 2014;9(8):910–26. Available from: http://www.pubmedcentral.nih.gov/articlerender.fcgi?artid=4166913&tool=pmcentrez&rendertype=abstract
67. Ved R, Scott K. Counseling Is a Relationship Not Just a Skill: Re-conceptualizing Health Behavior Change Communication by India's Accredited Social Health Activists. Glob Heal Sci Pract. 2020;8(3).
68. Perry HB, Zulliger R, Scott K, Javadi D, Gergen J, Shelley K, et al. Case Studies of Large-Scale Community Health Worker Programs: Examples from Afghanistan, Bangladesh, Brazil, Ethiopia, India, Indonesia, Iran, Nepal, Niger, Pakistan, Rwanda, Zambia, and Zimbabwe [Internet]. Washington, DC; 2017. Available from: http://www.mcsprogram.org/wp-content/uploads/2017/01/CHW-CaseStudies-Globes.pdf

For peer review only

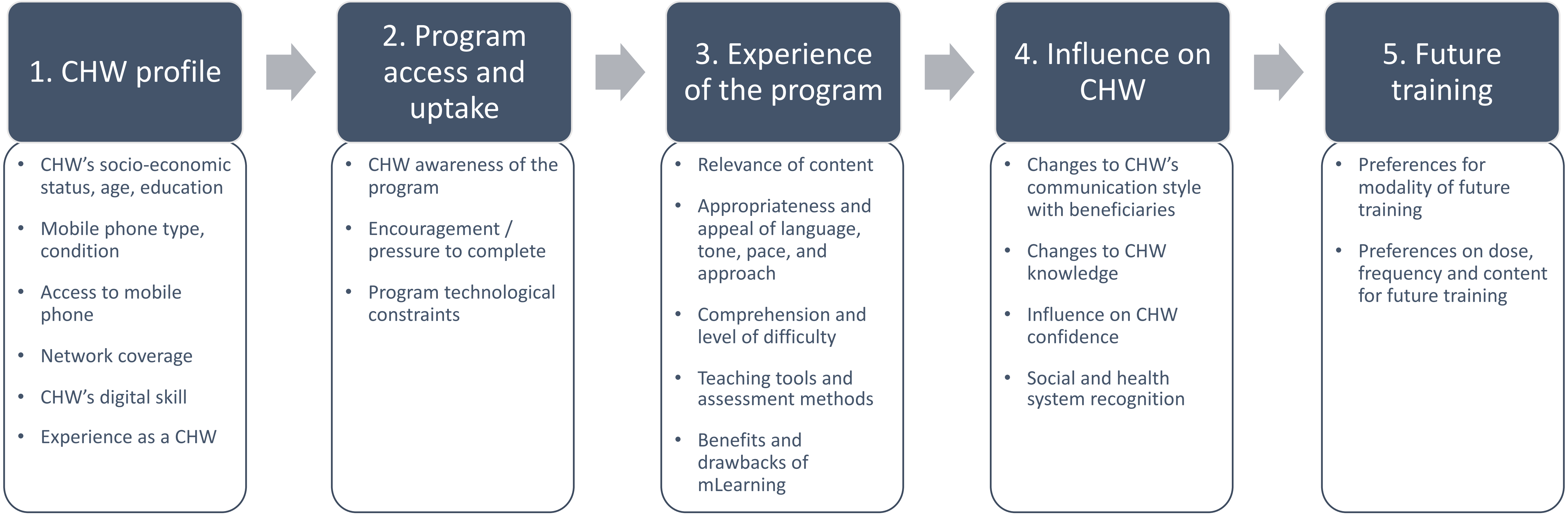
1. CHW profile
CHW's socio-economic status, age, education
Mobile phone type, condition
Access to mobile phone
Network coverage
CHW's digital skill
Experience as a CHW
2. Program access and uptake
CHW awareness of the program
Encouragement / pressure to complete
Program technological constraints
3. Experience of the program
Relevance of content
Appropriateness and appeal of language, tone, pace, and approach
Comprehension and level of difficulty
Teaching tools and assessment methods
Benefits and drawbacks of mLearning
4. Influence on CHW
Changes to CHW's communication style with beneficiaries
Changes to CHW knowledge
Influence on CHW confidence
Social and health system recognition
5. Future training
Preferences for modality of future training
Preferences on dose, frequency and content for future training

COREQ (COnsolidated criteria for REporting Qualitative research) Checklist

A checklist of items that should be included in reports of qualitative research. You must report the page number in your manuscript where you consider each of the items listed in this checklist. If you have not included this information, either revise your manuscript accordingly before submitting or note N/A.

Topic	Item No.	Guide Questions/Description	Reported on Page No.
**Domain 1: Research team and reflexivity**			
Personal characteristics			
Interviewer/facilitator	1	Which author/s conducted the interview or focus group?	
Credentials	2	What were the researcher's credentials? E.g. PhD, MD	
Occupation	3	What was their occupation at the time of the study?	
Gender	4	Was the researcher male or female?	
Experience and training	5	What experience or training did the researcher have?	
Relationship with participants			
Relationship established	6	Was a relationship established prior to study commencement?	
Participant knowledge of the interviewer	7	What did the participants know about the researcher? e.g. personal goals, reasons for doing the research	
Interviewer characteristics	8	What characteristics were reported about the inter viewer/facilitator? e.g. Bias, assumptions, reasons and interests in the research topic	
**Domain 2: Study design**			
Theoretical framework			
Methodological orientation and Theory	9	What methodological orientation was stated to underpin the study? e.g. grounded theory, discourse analysis, ethnography, phenomenology, content analysis	
Participant selection			
Sampling	10	How were participants selected? e.g. purposive, convenience, consecutive, snowball	
Method of approach	11	How were participants approached? e.g. face-to-face, telephone, mail, email	
Sample size	12	How many participants were in the study?	
Non-participation	13	How many people refused to participate or dropped out? Reasons?	
Setting			
Setting of data collection	14	Where was the data collected? e.g. home, clinic, workplace	
Presence of non-participants	15	Was anyone else present besides the participants and researchers?	
Description of sample	16	What are the important characteristics of the sample? e.g. demographic data, date	
Data collection			
Interview guide	17	Were questions, prompts, guides provided by the authors? Was it pilot tested?	
Repeat interviews	18	Were repeat inter views carried out? If yes, how many?	
Audio/visual recording	19	Did the research use audio or visual recording to collect the data?	
Field notes	20	Were field notes made during and/or after the inter view or focus group?	
Duration	21	What was the duration of the inter views or focus group?	
Data saturation	22	Was data saturation discussed?	
Transcripts returned	23	Were transcripts returned to participants for comment and/or	

For peer review only

Topic	Item No.	Guide Questions/Description	Reported on Page No.
		correction?	
**Domain 3: analysis and findings**			
Data analysis			
Number of data coders	24	How many data coders coded the data?	
Description of the coding tree	25	Did authors provide a description of the coding tree?	
Derivation of themes	26	Were themes identified in advance or derived from the data?	
Software	27	What software, if applicable, was used to manage the data?	
Participant checking	28	Did participants provide feedback on the findings?	
Reporting			
Quotations presented	29	Were participant quotations presented to illustrate the themes/findings? Was each quotation identified? e.g. participant number	
Data and findings consistent	30	Was there consistency between the data presented and the findings?	
Clarity of major themes	31	Were major themes clearly presented in the findings?	
Clarity of minor themes	32	Is there a description of diverse cases or discussion of minor themes?	

Developed from: Tong A, Sainsbury P, Craig J. Consolidated criteria for reporting qualitative research (COREQ): a 32-item checklist for interviews and focus groups. *International Journal for Quality in Health Care*. 2007. Volume 19, Number 6: pp. 349 – 357

Once you have completed this checklist, please save a copy and upload it as part of your submission. DO NOT include this checklist as part of the main manuscript document. It must be uploaded as a separate file.

For peer review only
